# Supplementary material for: A comprehensive structural analysis of the ATPase domain of human DNA topoisomerase II beta bound to AMPPNP, ADP, and the bisdioxopiperazine, ICRF193
Source: Structure. 2022 Aug 4;30(8):1129–1145.e3. doi: 10.1016/j.str.2022.05.009 (PMC9592559; doi:10.1016/j.str.2022.05.009)
Supplement: Document S1. Figures S1–S3 and Table S1 [file mmc1.pdf]

Structure, Volume *30*

Supplemental Information

A comprehensive structural analysis of the ATPase domain of human DNA topoisomerase II beta bound to AMPPNP, ADP, and the bisdioxopiperazine, ICRF193

Elise M. Ling, Arnaud Baslé, Ian G. Cowell, Bert van den Berg, Tim R. Blower, and Caroline A. Austin

Supplementary Information for Ling et al, 2022

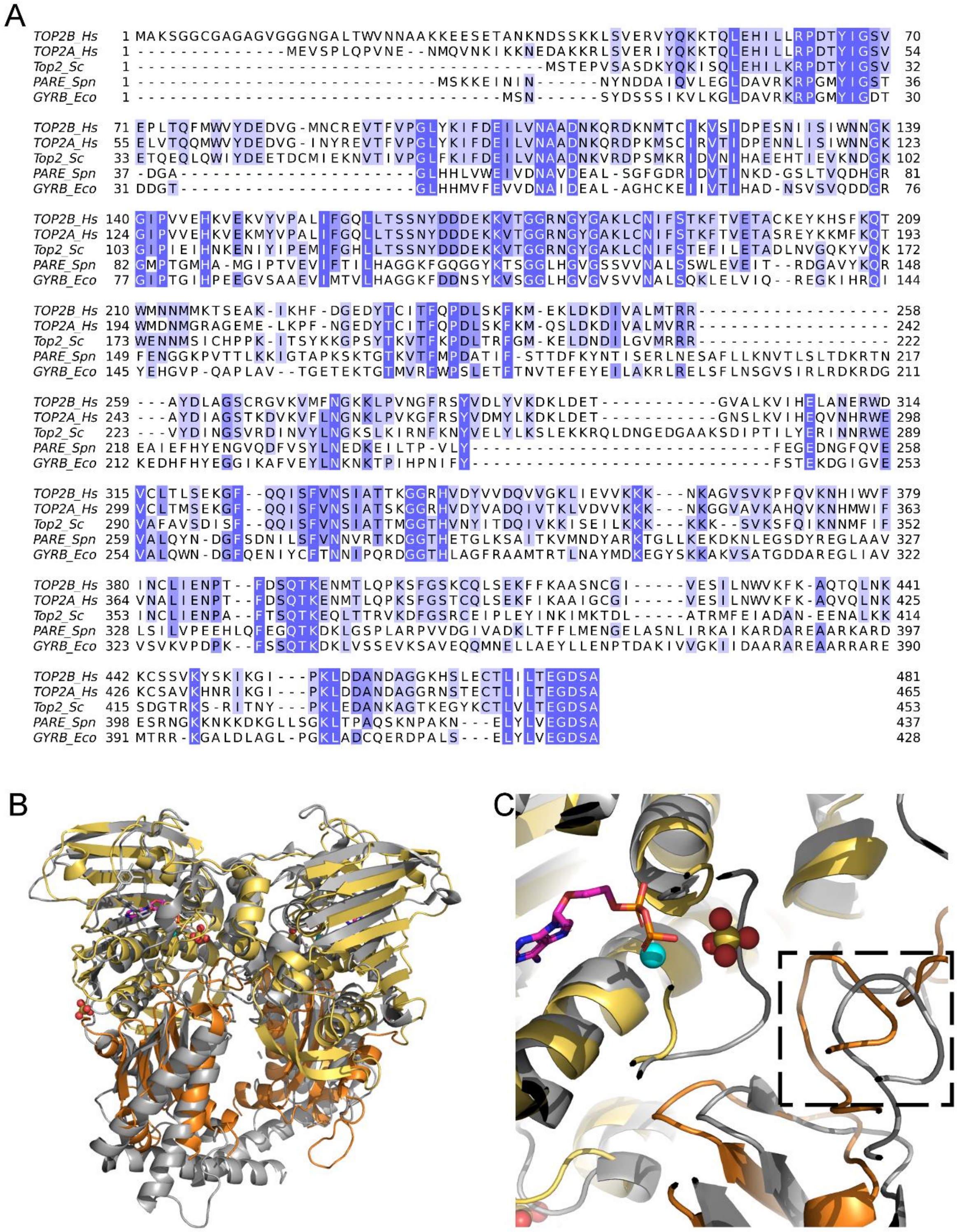

Supplementary Figure 1: Clustal Alignment and Crystal Structures of GyrB, Related to Figure 1

(**A**) Clustal alignment comparing Gyrase B, ParE with eukaryotic type II topoisomerases (human TOP2B, human TOP2A, yeast TOP2). (**B-C**) Superposition of Gyrase B (grey) (pdb 1KIJ) onto human TOP2B:ADP ATPase domain (yellow and orange). The transducer helices towards the end of the ATPase domain in GyrB are in a more open conformation compared to human TOP2B. Moreover, the QTK loop in GyrB (Panel C boxed region) is further from the active site in comparison to human TOP2B.

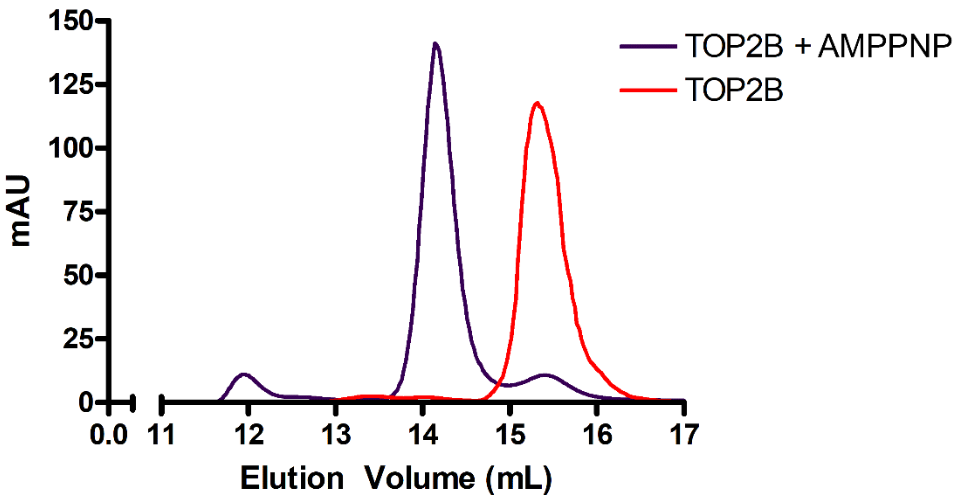

Supplementary Figure 2: Analytical Gel Filtration of the ATPase Domain of Human TOP2B, Related to Figure 1

Analytical gel filtration of TOP2B (45-444) in the absence (red) and presence (purple) of AMPPNP. TOP2B protein injected onto a Superdex 200 Increase 10/300 GL column with 20 mM Tris HCl (pH 8.0), 150 mM NaCl. Blue line indicates UV absorbance. In the absence of AMPPNP, TOP2B elutes at ~15.3 mL corresponding to an apparent Mw of 47 kDa, equivalent to the theoretical size (45 kDa), whereas upon addition of AMPPNP, the protein elutes earlier at ~14.1 mL, corresponding to an apparent Mw of 78 kDa.

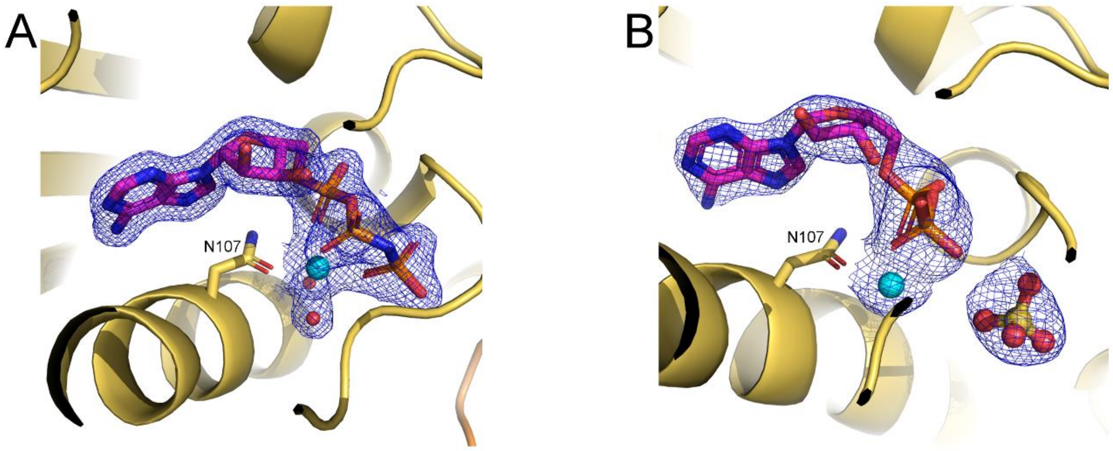

Supplementary Figure 3: Omit Maps for Bound Nucleotides, Related to Figure 2 (A) and Figure 3 (B)

The 2 Fo – Fc maps electron density maps for the 1.9 Å resolution AMPPNP complex (**A**), and the 2.6 Å resolution ADP complex (**B**) represented by blue mesh. The maps are contoured at levels of 1.5 σ and carved at 1.6 Å. Mg^{2+} shown in cyan and two water molecules shown in A as red spheres and sulphate ion represented by red and yellow spheres in B. Residue N107 shown as sticks.

Supplementary Table 1: Summary of selected homologous PDB entries relevant to this study and related to Figure 1B, Figure 5, Figure 6, STAR Methods and Supplemental Figure 1.

Protein Source	PDB code	Resolution (Å)	Conformation	Nucleotide	PMID	Reference
GyrB43 *E.coli*	N/A	2.5	closed	AMPPNP	1646964	Wigley et al, 1991
GyrB43 *E.coli*	4WUB	1.75	restrained	AMPPNP	25849408	Hearnshaw et al. 2015
GyrB43 *E.coli*	4PU9	2.4	semi-open Pre-hydrolysis	ADP.BeF3	25202966	Stanger et al. 2014
GyrB43 *E.coli*	4PRX	1.8	open Post-hydrolysis	ADP.Pi	25202966	Stanger et al. 2014
GyrB43 *E.coli*	4PRV	2.0	Closed product	ADP	25202966	Stanger et al. 2014
GyrB43 *E.coli*	1EI1	2.3	closed	AMPPNP	10734094	Brino et al. 2000
GyrB43 *E.coli*	1KIJ	2.3	open	(novobiocin and formic acid)	11850422	Lamour et al. 2002
ParE *E.Coli*	1S16	2.1	closed	AMPNP	15105144	Bellon et al. 2004
ParE *S. pneumoniae*	5J5P	2.83	closed	AMPNP	29968711	Laponogov, et al, 2018
ParE S. pneumoniae	5J5Q	2.83	closed	AMPNP	29968711	Laponogov, et al, 2018
Archaeal GHL enzyme topoisomerase VI topoVI-B *S. shibatae*	1MU5	2.0	relaxed	Apo	12505993	Corbett & Berger 2003
topoVI-B *S. shibatae*	1MX0	2.3	restrained	AMPNP	12505993	Corbett & Berger 2003
topoVI-B *S. shibatae*	1Z5B	2.0	restrained	$ADP.AIF_4^-$	15939019	Corbett & Berger 2005
topoVI-B *S. shibatae*	1Z5C	2.2	restrained	ADP.Pi	15939019	Corbett & Berger 2005
topoVI-B S. shibatae	1Z5A	2.2	Restrained (dimer)	ADP	15939019	Corbett & Berger 2005
topoVI-B S. shibatae	1Z59	2.1	Relaxed (monomer)	ADP	15939019	Corbett & Berger 2005
TOP2 *S.Cerevisiae*	1PVG	1.8	restrained	AMPPNP	12963818	Classen et al. 2003
TOP2A *H. sapiens*	1ZXM	1.87	restrained	AMPPNP	16100112	Wei et al. 2005
TOP2A *H. sapiens*	1ZXN	2.51	open	ADP	16100112	Wei et al. 2005
TOP2A *H. sapiens*	4R1F	-	open	ADP.SO4	25202966	Stanger et al. 2014
TOP2 *S.Cerevisiae*	4GFH	4.41		AMPPNP	23022727	Schmidt, Osheroff and Berger 2012
