# Supplementary material for: A comprehensive structural analysis of the ATPase domain of human DNA topoisomerase II beta bound to AMPPNP, ADP, and the bisdioxopiperazine, ICRF193
Source: Structure. 2022 Aug 4;30(8):1129–1145.e3. doi: 10.1016/j.str.2022.05.009 (PMC9592559; doi:10.1016/j.str.2022.05.009)
Supplement: Document S2. Article plus supplemental information [file mmc2.pdf]

Structure

Article

A comprehensive structural analysis of the ATPase domain of human DNA topoisomerase II beta bound to AMPPNP, ADP, and the bisdioxopiperazine, ICRF193

Graphical abstract

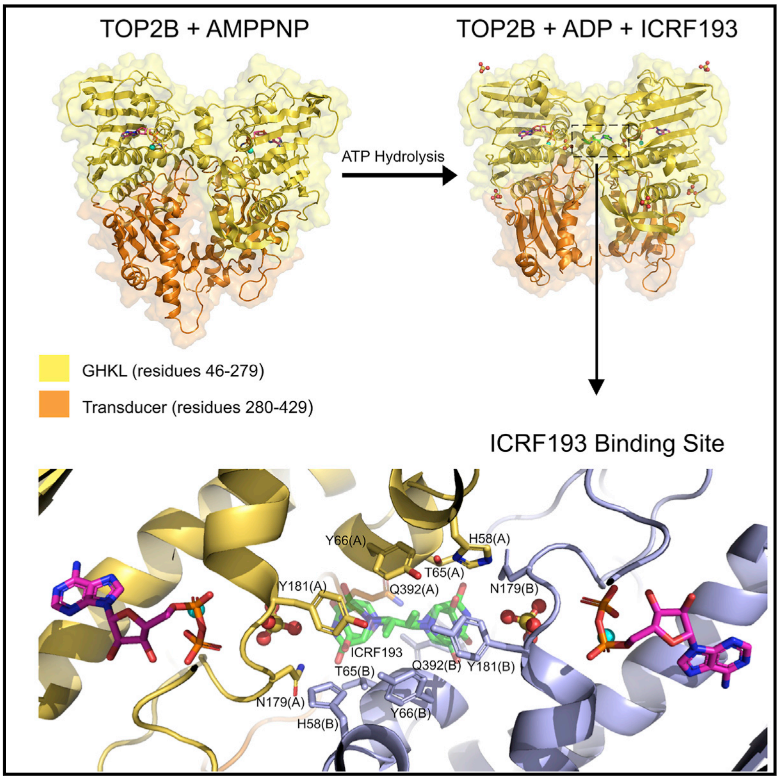

Highlights

- Three structures of the TOP2B ATPase domain bound to AMPPNP, ADP, or ICRF193
- The QTK loop in the ADP complex is further from the active site
- An SO_4 ion is in place of the ATP hydrolysis product, P_i
- Biochemical data show the N-terminal strap reduces the ATPase hydrolysis activity

Authors

Elise M. Ling, Arnaud Baslé, Ian G. Cowell, Bert van den Berg, Tim R. Blower, Caroline A. Austin

Correspondence

timothy.blower@durham.ac.uk (T.R.B.), caroline.austin@ncl.ac.uk (C.A.A.)

In brief

Ling et al. report the crystal structures of the human TOP2B ATPase domain bound to AMPNP or ADP or ADP and ICRF193. ICRF193 is a bisdioxopiperazine that inhibits DNA topoisomerase II activity, key residues bound by ICRF193 are identified. Conformational changes within the enzyme during ATP hydrolysis are reported.

Ling et al., 2022, Structure *30*, 1129–1145
August 4, 2022 © 2022 The Author(s). Published by Elsevier Ltd.
https://doi.org/10.1016/j.str.2022.05.009

CellPress

Structure

CellPress
OPEN ACCESS

Article

A comprehensive structural analysis of the ATPase domain of human DNA topoisomerase II beta bound to AMPPNP, ADP, and the bisdioxopiperazine, ICRF193

Elise M. Ling,[1] Arnaud Baslé,[1] Ian G. Cowell,[1] Bert van den Berg,[1] Tim R. Blower,[2,*] and Caroline A. Austin[1,3,*]
[1]Biosciences Institute, Newcastle University, Newcastle upon Tyne NE2 4HH, UK
[2]Department of Biosciences, Durham University, South Road, Durham DH1 3LE, UK
[3]Lead contact
*Correspondence: timothy.blower@durham.ac.uk (T.R.B.), caroline.austin@ncl.ac.uk (C.A.A.)
https://doi.org/10.1016/j.str.2022.05.009

SUMMARY

Human topoisomerase II beta (TOP2B) modulates DNA topology using energy from ATP hydrolysis. To investigate the conformational changes that occur during ATP hydrolysis, we determined the X-ray crystallographic structures of the human TOP2B ATPase domain bound to AMPPNP or ADP at 1.9 Å and 2.6 Å resolution, respectively. The GHKL domains of both structures are similar, whereas the QTK loop within the transducer domain can move for product release. As TOP2B is the clinical target of bisdioxopiperazines, we also determined the structure of a TOP2B:ADP:ICRF193 complex to 2.3 Å resolution and identified key drug-binding residues. Biochemical characterization revealed the N-terminal strap reduces the rate of ATP hydrolysis. Mutagenesis demonstrated residue E103 as essential for ATP hydrolysis in TOP2B. Our data provide fundamental insights into the tertiary structure of the human TOP2B ATPase domain and a potential regulatory mechanism for ATP hydrolysis.

INTRODUCTION

Type II DNA topoisomerases are enzymes that use the energy from ATP hydrolysis to regulate DNA topology, such as relieving DNA supercoiling during transcription and decatenation of replication products. Type II DNA topoisomerases are essential for cell viability. The type II enzymes comprise four discrete structural domains revealed by three interdomain protease sensitive sites, A, B, and C (Austin et al., 1995) (Figure 1A). The structural domains are evolutionarily conserved, sharing sequence similarity (Figure 1B) and contain highly conserved functional motifs (Figure 1A) (Austin et al., 1993). The N-terminal ATPase domain of type II topoisomerases is the most highly conserved domain and is homologous to that of the bacterial gyrase B subunit (GyrB) (Ali et al., 1993) (Figure S1). The N-terminal half of the ATPase domain contains the ATP-binding site and is directly involved in clamp closure, undergoing dimerization upon ATP binding. The ATP-binding fold within this region is known as the Bergerat fold, which is common to the GHKL-type ATPases (Corbett and Berger 2004; Dutta and Inouye 2000). The C-terminal half of the ATPase domain forms the transducer domain, which connects the ATPase domain to the enzyme core (Oestergaard et al., 2004). The transducer domain relays the signals of ATP binding and hydrolysis between the ATPase domain and the breakage-rejoining core via conformational changes in the enzyme (Classen et al. 2003; Schmidt et al. 2012; Vanden Broeck et al., 2021). The central breakage-rejoining domain or core domain is homologous to the bacterial gyrase A subunit (GyrA) and is made up of two structural domains: a metal-binding domain, which has homology with primases so has been termed the TOPRIM domain; and a DNA-cleavage domain, which provides the site of covalent attachment to DNA via the catalytic tyrosine. The C-terminal domain of type II topoisomerases is the least conserved domain and contains the nuclear localization signals and many phosphorylation sites. *Saccharomyces cerevisiae* has one isoform of topoisomerase II, while vertebrates, including humans, have two type II isoforms, TOP2A and TOP2B, with differing roles. TOP2A is essential for cell division, while TOP2B is required in post-mitotic cells (Austin et al., 2021) as well as dividing cells and is essential for development of the nervous system (Yang et al., 2000; Lyu and Wang 2003) and the immune system (Broderick et al., 2019). Human TOP2B possesses an alternative splice variant in the ATPase domain, where an additional five amino acids (T-L-F-D-Q) encoded by 15 nucleotides are inserted into the mRNA via differential splicing after valine 23 (Davies et al. 1993) (Figure 1A). Both TOP2B isoforms are expressed in human tissues, as detailed from RNA sequencing (RNA-seq) data on the genotype-tissue expression (GTEx) portal (Aguet et al., 2020).

Type II DNA topoisomerases alter the topology of DNA by strand passage. One DNA helix, termed the gate helix (G-DNA), is transiently cleaved, and a second helix, termed the transported helix (T-DNA), is passed through the enzyme-bridged break (Roca and Wang 1992) (Figure 1C). Strand

This is an open access article under the CC BY license (http://creativecommons.org/licenses/by/4.0/).

A

B

C

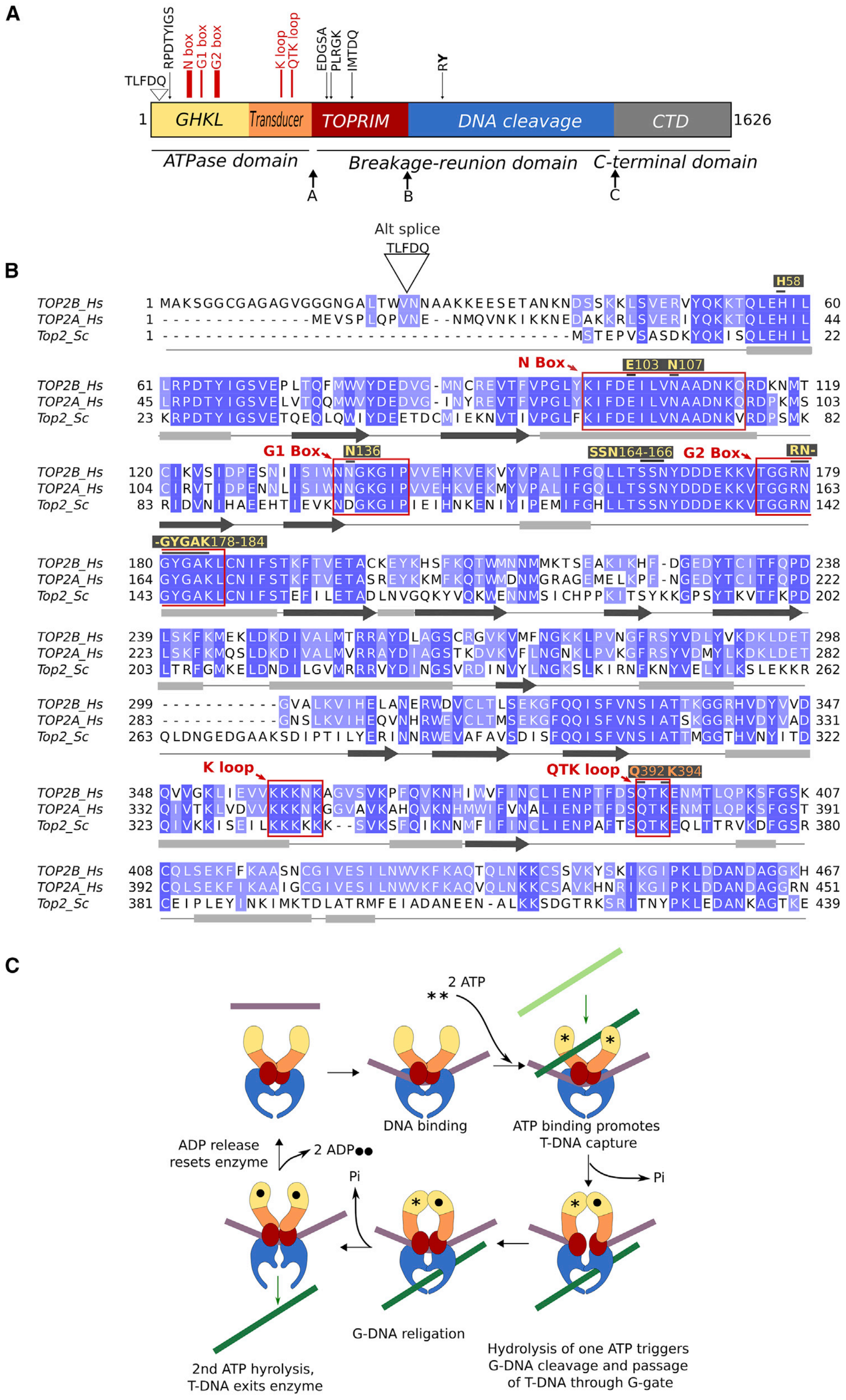

(legend on next page)

CellPress
OPEN ACCESS

passage requires ATP hydrolysis, although DNA cleavage may occur in its absence. The ATPase domain binds and hydrolyzes ATP in a DNA-dependent manner (Lindsley and Wang 1993; Olland and Wang 1999; Hammonds and Maxwell 1997; Gardiner et al., 1998). Using yeast TOP2 and pre-steady-state kinetic techniques, it has been shown that each protein dimer binds two ATP molecules, one of which is hydrolyzed rapidly, while hydrolysis of the second ATP molecule is rate limited by the release of P_i or ADP (Harkins et al. 1998; Baird et al., 2001). The DNA transport event occurs between hydrolysis of the first and second ATP molecules. However, binding of just one ATP molecule is sufficient to induce a conformational change across the dimer (Baird et al., 1999; Skouboe et al., 2003).

The catalytic cycle of topoisomerases has been targeted by a variety of antibacterial and anticancer drugs. One class of anticancer agents are the bisdioxopiperazines (ICRF187 and ICRF193), which are catalytic inhibitors of human topoisomerase II (Lee et al. 2017). Kinetic studies have demonstrated that the bisdioxopiperazines are non-competitive inhibitors of type II topoisomerases and do not directly compete for the ATP-binding site (Morris et al. 2000). The bisdioxopiperazines associate with a nucleotide-bound dimerized state of the ATPase domain and trap the enzyme around DNA by stabilizing the N-terminal dimer interaction formed by ATP binding, blocking enzyme turnover (Roca et al., 1994). ICRF187 (dexrazoxane) is used therapeutically for the prevention of anthracycline-induced cardiotoxicity, while ICRF193 is the most potent bisdioxopiperazine against topoisomerase II (Hasinoff et al., 1995; Hasinoff et al. 2020; Jirkovská et al., 2021). As the only topoisomerase II isoform in postmitotic cardiac cells is human TOP2B, the ATPase domain of human TOP2B is the physiological target of ICRF187 (Capranico et al., 1992; Atwal et al., 2019). Moreover, bisdioxopiperazines inhibit the catalytic activity of the two human isoforms differentially (Shapiro and Austin 2014), and the human isoforms are more sensitive to ICRF187 than *S. cerevisiae* topoisomerase II (Lee et al. 2017), providing the rationale for solving the structure of human TOP2B ATPase domain. TOP2A mutations that confer drug resistance to the bisdioxopiperazines have been reported (D48N, Y50F, R162Q, Y165S, L169F) (Wessel et al. 1999, 2002; Sehested et al., 1998; Patel et al., 2000). These residues are conserved between human TOP2A and TOP2B (Figure 1B), and, when the corresponding residue was mutated in TOP2B (D64N, Y66F, R178Q, Y181S, and L185F), except for R178Q, all mutants were resistant to ICRF187 compared with wild-type TOP2B (Deng et al., 2014). In addition, a site-directed mutation that reduced ATPase activity (S165R) (West et al., 2002) and a random mutation that conferred drug resistance to mAMSA (G465D) (Gilroy et al., 2006) have been reported. Furthermore, the mutation H58Y (Lam et al. 2017; Hiraide et al., 2020) has been reported in two patients exhibiting developmental delay characteristic of autism.

As DNA topoisomerase II is a large protein of 180 kDa, structural studies have utilized individual domains. Solving the structure of domains of type II topoisomerases has provided a detailed understanding of the key aspects of the molecular mechanisms employed by these enzymes. Domains have been crystallized from a wide range of species (Table S1). Key functional motifs are conserved between species (Figures 1A, 1B, and S1A); however, there are important differences, such as a longer N-terminal strap in the human type II topoisomerases, with TOP2B being 14 residues longer at the N-terminal end than TOP2A. Recently position 28 has been reported to be an evolutionarily positively selected site (Moreira et al., 2022). The ATPase domain from DNA gyrase and archaeal topoisomerase VI (Table S1) has been solved in multiple conformations representing each step of the ATP turnover cycle (Wigley et al., 1991; Hearnshaw et al., 2015; Stanger et al., 2014; Brino et al., 2000; Lamour et al., 2002; Corbett et al., 2005; Corbett and Berger 2003). The core domain of yeast TOP2 (Berger et al., 1996; Schmidt et al., 2010) has been solved as well as the ATPase domain of yeast TOP2 in the presence and absence of the bisdioxopiperazine ICRF187 (Classen et al. 2003). A full-function tailless structure comprising the ATPase and the core domain of yeast TOP2 (Schmidt et al. 2012) has been solved and revealed a new control mechanism for ATPase activity that links the transducer region of the ATPase domain and the G strand (Table S1). For the human proteins, the core domain of TOP2A (Wendorff et al., 2012) and the ATPase domain of TOP2A (Wei et al., 2005) have been solved. In addition, a cryoelectron microscopy (cryo-EM) structure of full-length TOP2A has recently been published (Vanden Broeck et al., 2021), and this enabled the allosteric interactions between the ATPase domain and the TOPRIM domain to be analyzed.

Regarding human TOP2B, the structure of the core domain has been reported (Wu et al., 2011), but to date the ATPase domain of human TOP2B has not been published. Since human TOP2B is the target for bisdioxopiperazines, we set out to crystallize and solve the structure of the ATPase domain of TOP2B bound to nucleotides and drug. We report two structures for

Figure 1. Domain arrangement and mechanism of type II topoisomerases

(A) Schematic representation of the domain arrangement of type II topoisomerases with the alternative exon (TLFDQ) for human TOP2B indicated. Sites A, B, and C represent proteolytically sensitive sites in TOP2B. Five motifs conserved in all type II topoisomerases are shown in black and the key regions for ATPase activity are indicated in red.

(B) Clustal alignment of human TOP2B, human TOP2A, and *S. cerevisiae* ATPase domain. The N, G1, and G2 boxes are indicated as well as the K loop and the QTK loop. Residues involved in binding ATP are indicated and labeled in color according to (A). The secondary structure for TOP2B is shown, gray boxes for alpha helices and black arrows for beta sheets.

(C) Catalytic mechanism of type II topoisomerase enzymes (adapted from Schmidt et al. 2012). In the absence of bound G-DNA, the enzyme is in an open conformation with the ATPase (yellow and orange), red and blue domains separated. When the G-DNA (purple) binds, a conformational change occurs that brings the active-site tyrosines into their attack positions. Next, ATP (denoted by an asterisk [*]) binds and the ATPase domains dimerize, causing a further conformational change in the enzyme. During ATPase domain dimerization, a second DNA duplex, T-DNA (green) is captured, and pushed into the central cavity of the enzyme, and through the cleaved G-DNA (DNA gate). Once the T-DNA has entered the central cavity, the religation of the G-DNA can occur. A linked conformational change opens the C-gate through which the T-DNA exits as described in the two-gate model (Berger et al., 1996; Roca and Wang 1994; Wang 1998). ATP hydrolysis resets the enzyme for another round of catalysis.

the ATPase domain of human TOP2B (45–444), one in the presence of the non-hydrolysable ATP analogue AMPPNP and another in the presence of ADP, using data to 1.9 Å and 2.6 Å respectively. We find that residues Q392 and K394 within the QTK loop move by 3.8 Å and 2.5 Å respectively, to open the active site for release of the ATP hydrolysis product P_i. To define interactions between the clinical target of bisdioxopiperazines, we report the structure of the ATPase domain of human TOP2B (45–444) in complex with ICRF193 to 2.3 Å and identify key residues in TOP2B that are involved in drug binding. The availability of these structures provides a vital resource for future drug design and analysis of specific drug-resistant mutations that may arise. We also present activity data on the full-length TOP2B ATPase domain (1–444) and the alternative splice variant (1–449), where we find the N-terminal strap to be inhibitory to ATP hydrolysis. Moreover, we biochemically demonstrate that the residue E103 is the catalytic base for ATP hydrolysis in TOP2B and that mutating this to an alanine abolishes ATP hydrolysis activity.

RESULTS

Structure of the human TOP2B ATPase domain bound to AMPPNP

The present study aimed to gain insights into the tertiary structure of the ATPase domain of human TOP2B and to determine whether ATP hydrolysis results in conformational changes within the enzyme. As such, we purified and crystallized the TOP2B ATPase domain (45–444) in the presence of two different nucleotides: AMPPNP and ADP. The ATPase domain of TOP2B in complex with AMPPNP was solved to 1.9 Å resolution (PDB: 7QFO) (Figure 2A), by molecular replacement using the human TOP2A ATPase domain structure as a search model (Wei et al., 2005). The final TOP2B:AMPPNP model contains residues 46–429 and was refined to a R_{work} factor of 19.2% and an R_{free} of 23.2% (Table 1). The asymmetric unit contains one copy of the protein, and the biological dimer was generated by applying crystallographic symmetry. Analytical gel filtration of TOP2B (45–444) in the presence and absence of AMPPNP (Figure S2) further confirms the presence of a dimer, as well as interactions between monomers determined by PISA (Krissinel and Kim 2007). The interface area of the dimer represents ~13% of the total monomer surface, indicating significant interactions, which include 24 hydrogen bonds and 18 salt bridges between the two monomers. The electron density corresponding to the final 15 residues at the C terminus of this domain was too weak to model, hence residues 430–444 were not modeled in the final structure.

The TOP2B ATPase domain dimerizes in the presence of AMPPNP, giving rise to the ATP-restrained state (Figure 2A) previously described for bacterial DNA gyrase (Stanger et al., 2014). A heart-shaped dimer is formed with a central cavity that is 31 Å high and 23 Å wide, the dimensions of which cannot easily accommodate a DNA duplex (Figure 2A). Each TOP2B ATPase protomer folds into two discrete structural modules: the N-terminal GHKL domain and the C-terminal transducer domain (colored yellow and orange respectively in Figure 2A). The GHKL domain of human TOP2B (residues 46–279) comprises an eight-stranded antiparallel β sheet floor and four α-helical walls, giving rise to the Bergerat fold (Corbett and Berger 2004; Dutta and Inouye 2000; Bergerat et al., 1997). The Bergerat fold typically consists of four conserved motifs: the N box, G1 box, G2 box, and G3 box (Figure 1B). The N box of TOP2B is located between residues 99 and 113 and contains a conserved asparagine, N107, that coordinates the catalytic Mg^{2+} ion and contains a conserved glutamate, E103, which acts as a general base to promote nucleophilic attack on the γ phosphate of ATP using a water molecule (Dutta and Inouye 2000; Bergerat et al., 1997) (Figures 2B–2D). The G1 box is located between residues 136 and 142 and houses a conserved asparagine, N136, which hydrogen bonds with the adenine ring of AMPPNP. Residues 175–185 comprise the G2 box, previously described as a Walker A motif (Wessel et al. 1999, 2002; Walker et al., 1982). Moreover, the conserved lysine residue K168 in human TOP2A (K184 in TOP2B) located in the G2 box has been shown to undergo acetylation, which can regulate ATP hydrolysis (Bedez et al., 2018). The G3 box is absent from the linear sequence and tertiary structure of the eukaryotic type II topoisomerases. The transducer domain (residues 280–429) consists of a four-stranded mixed β sheet backed by three α helices and contains the switch lysine (K394 in TOP2B), which is absolutely conserved in all type II topoisomerases (Smith and Maxwell 1998; Wei et al., 2005). The switch lysine is part of a highly conserved QTK loop, residues 392–394 (Figure 1B), that extends into the ATP-binding pocket. Toward the C-terminal end of the structure is the K loop, residues 358–362 (KKKNK) in TOP2B, which couples DNA binding to ATP hydrolysis and strand passage activity (Schmidt et al. 2012).

As anticipated from prior ATP-restrained structures (Stanger et al., 2014; Wei et al., 2005; Classen et al. 2003), the active site of the TOP2B ATPase domain in the presence of AMPPNP is sequestered from solvent and poised for ATP hydrolysis. Side chains of residues in the conserved N box, SSN motif, G2 box, and the QTK loop involved in AMPPNP binding are represented as green lines in Figure 2B or sticks in 2C and D. Residues that contribute binding to AMPPNP via main-chain bonding are located in the conserved G2 box and are represented as blue lines in Figure 2B or spheres in 2C and D. The AMPPNP-binding site is predominantly composed of residues from the GHKL domain, with only two transducer domain residues (Q392 and K394 from the QTK loop) making direct contacts with AMPPNP. To complete the binding site, the N-terminal strap motif from one monomer reaches across the dimer interface to form the ATP lid of the partner monomer. However, the complete extent of nucleotide sequestering is not observed in our structure as the initial 44 amino acids are absent. The adenine ring of AMPPNP is held in place through direct hydrogen bonding with the carbonyl of N136, and stabilization of the ribose sugar is achieved via hydrogen bonding to the hydroxyl group of S165. Hydrogen bonds are made to the α and β phosphate groups of AMPPNP by the side chains of residues N107 and K184, and S164 and N166 respectively. Moreover, the main chain of A183 contributes a hydrogen bond to the α phosphate group (Figure 2B). The side chains of Q392 and K394 from the QTK loop of the transducer domain hold the γ phosphate firmly in place, with the switch lysine, K394, forming a salt bridge that is believed to stabilize the transition state of the hydrolysis reaction (Smith and Maxwell 1998).

CellPress
OPEN ACCESS

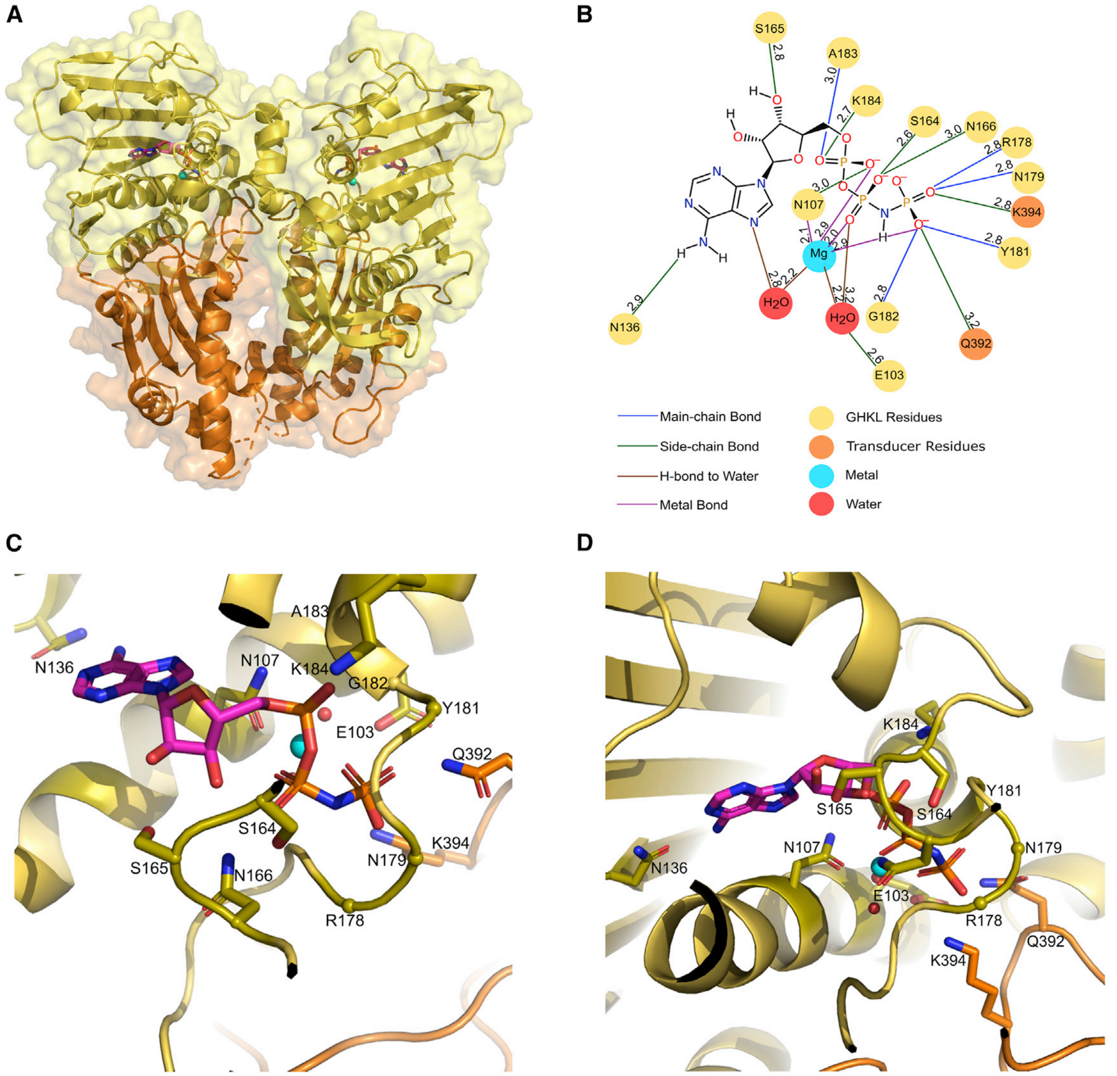

Figure 2. Overall structure and binding sites of the ATPase domain of human TOP2B bound to AMPPNP

(A) The GHKL and transducer domains of TOP2B are colored yellow and orange, respectively. Mg^{2+} ions colored in cyan. AMPPNP colored by atom: carbon, pink; nitrogen, blue; oxygen, red; and phosphate, orange. The surface representation is also shown.

(B) 2D diagram showing amino acid residues in TOP2B that interact with AMPPNP. Distances shown in angstroms. Blue lines represent a main-chain hydrogen bond, green lines represent a side-chain hydrogen bond, brown lines represent a hydrogen bond with water, and a purple line indicates a hydrogen bond with a metal ion.

(C and D) Two different views of the AMPPNP-binding site of TOP2B colored according to (A). The side chains of residues involved in hydrogen bonding to the nucleotide are shown in stick representation and colored by atom. Residues that bind nucleotides via main-chain bonding are represented as spheres. Two water molecules are shown as red spheres and Mg^{2+} ions as cyan spheres. Residue numbering according to the TOP2B (1–444) sequence.

The γ phosphate also forms an additional four hydrogen bonds with the main chain of residues R178, N179, Y181, and G182. The active site Mg^{2+} ion is coordinated by the conserved asparagine, N107, two water molecules, and all three phosphates of AMPPNP to form a distorted octahedral metal ion co-ordination shell (Wei et al., 2005; Classen et al. 2003) (Figure 2B). The key catalytic residue in TOP2B, E103, forms a hydrogen bond to one of the two water molecules coordinated to Mg^{2+}, which activates the water to allow nucleophilic attack on the γ phosphate of ATP. The electron density supporting AMPPNP binding is shown in Figure S3A.

Structure of the human TOP2B ATPase domain bound to ADP

To determine the difference in structure between pre- and post-ATP hydrolysis, we solved the structure of the TOP2B ATPase domain in complex with ADP (PDB: 7QFN) (Figure 3A). The structure was solved to 2.6 Å by molecular replacement using the TOP2B:AMPPNP structure as a search model. The final ADP-bound model contains residues 47–422 with missing loops between residues 169 and 173 and 356 and 366, where the K loop resides. The ADP-bound model was refined to an R_{work} factor of 20.7% and an R_{free} of 25.1% (Table 1). For both the

Table 1. Data statistics and refinement details for human TOP2B ATPase domain in the presence of AMPPNP, ADP, and ADP:ICRF193

	AMPPNP	ADP	ADP:ICRF193
Data statistics[a]			
Beamline	I03	I03	I03
Date	06/05/21	07/07/21	25/02/22
Wavelength (Å)	0.980	0.979	1.000
Resolution (Å)	59.41–1.90 (1.94–1.90)	89.22–2.62 (2.74–2.62)	53.66–2.30 (2.38–2.30)
Space group	$P4_12_12$	P 3 2 1	P 3 2 1
Unit-cell parameters			
a (Å)	84.02	103.02	103.40
b (Å)	84.02	103.02	103.40
c (Å)	127.09	66.69	67.02
$\alpha = \beta = \gamma$ (°)	90.00	90.00 = 90.00 = 120.00	90.00 = 90.00 = 120.00
Unit-cell volume ($Å^3$)	897,205	613,005.13	620,549.06
Solvent content (%)	50	45.9	45.8
No. of measured reflections	1,759,478 (117,137)	256,943 (32,178)	357,987 (36,121)
No. of independent reflections	36,646 (2,300)	12,596 (1,513)	18,666 (1,821)
Completeness (%)	100.0 (99.9)	100.0 (100.0)	99.8 (100)
Redundancy	48.0 (50.9)	20.4 (21.3)	19.2 (19.8)
$CC_{1/2}$ (%)	1.000 (0.568)	0.997 (0.780)	0.996 (0.565)
$\langle I \rangle / \langle \sigma(I) \rangle$	23.3 (1.2)	9.0 (1.7)	11.0 (1.5)
Refinement statistics[a]			
R_{work} (%)	19.30	20.70	19.70
R_{free} (%)	23.20	25.10	25.93
No. of non-H atoms			
No. of protein, atoms	3,044	2,886	2,944
No. of solvent atoms	99	13	50
No. of ligand atoms	32	38	41
RMSD from ideal values			
Bond angle (°)	1.73	1.66	1.82
Bond length (Å)	0.01	0.01	0.01
Average B factor ($Å^2$)			
Protein	44	60	44
Solvent	41	48	36
Ligand	32	50	46
Ramachandran plot,[b] residues in			
Most favored regions (%)	96.8	93.8	97.2
Allowed regions (%)	3.2	5.6	2.2
Disallowed regions (%)	0.0	0.6	0.6

5% of the randomly selected reflections excluded from refinement.
[a]Values in parentheses are for the highest-resolution shell.
[b]Calculated using MOLPROBITY.

TOP2B:AMPPNP and TOP2B:ADP models, the statistics are in line with published models of similar resolution and were confirmed during validation. Similar to the TOP2B:AMPPNP model, the ADP-bound structure has one copy of the protein per asymmetric unit and the biological dimer was confirmed by PISA (Krissinel and Henrick, 2007). The interface area of the dimer represents ~11% of the total monomer surface, indicating significant interactions, which include 34 hydrogen bonds and six salt bridges between the two monomers.

The ADP structure is composed of a virtually unchanged GHKL domain (Figure 3A) compared with the AMPPNP structure, with the nucleotide and most of the active-site residues in the same conformation (Figures 3B–3D). In addition, the ligand-binding site contains one bound ADP molecule per monomer. The adenine ring, ribose sugar, and α phosphate of ADP make the same set of residue contacts as in the AMPPNP complex, despite the sugar pucker differing. Similarly, S164 and N166, which hydrogen bond with the β phosphate of AMPPNP, also bind with the β phosphate of ADP, although the β phosphate of ADP has an additional contact to G180, which does not occur in the AMPPNP structure. As the γ phosphate is lost during ATP hydrolysis, R178 and N179, which were

CellPress
OPEN ACCESS

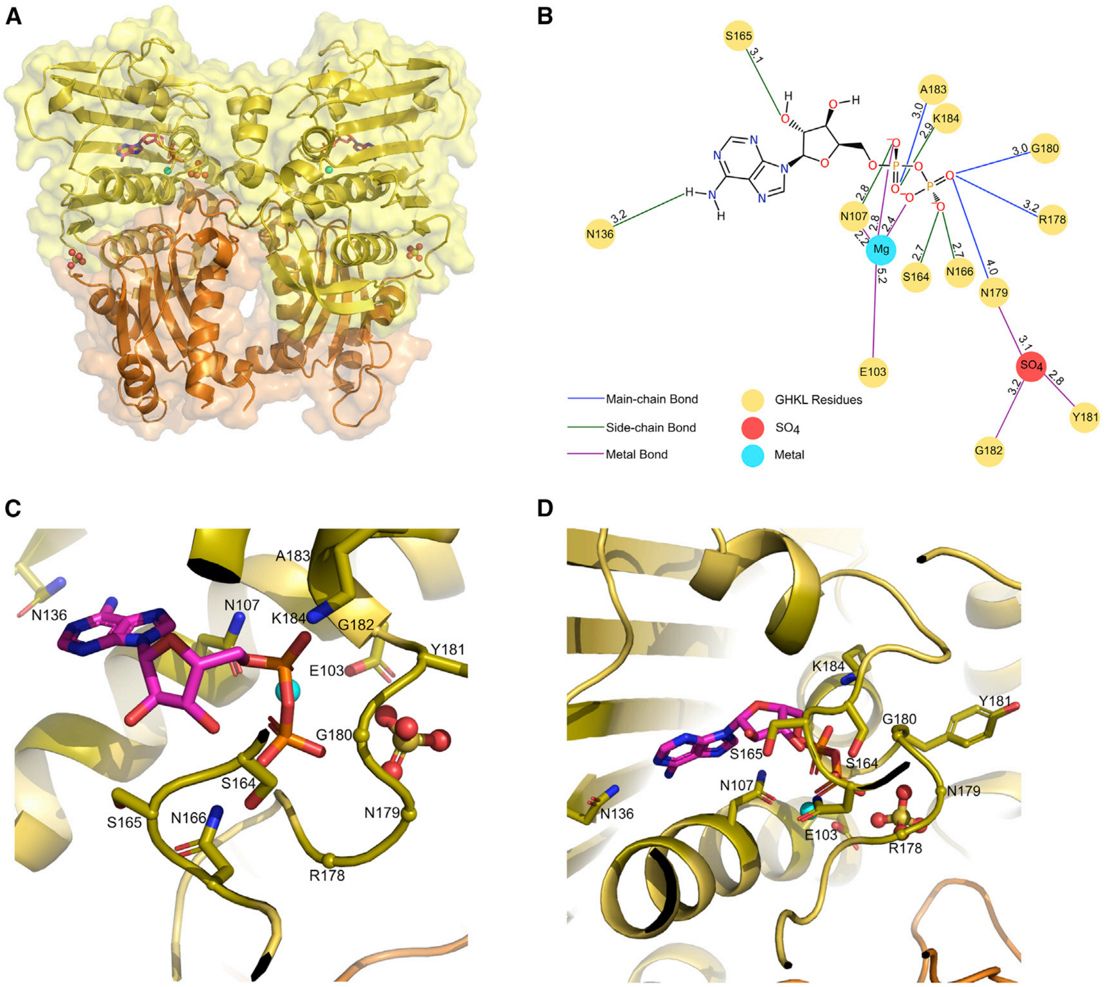

Figure 3. Overall structure and binding sites of the ATPase domain of human TOP2B bound to ADP

(A) The GHKL and transducer domains of TOP2B are colored yellow and orange respectively. ADP colored by atom: carbon, pink; nitrogen, blue; oxygen, red; and phosphate, orange. The surface representation is also shown. Mg^{2+} ions colored in cyan. Sulfate (SO_4) is represented by oxygen in red spheres and sulfur in yellow spheres.

(B) 2D diagram showing amino acid residues in TOP2B that interact with ADP. Distances shown in angstroms. Blue lines represent a main-chain hydrogen bond, green lines represent a side-chain hydrogen bond, brown lines represent a hydrogen bond with water, and a purple line indicates a hydrogen bond with a metal ion.

(C and D) Two different views of the ADP-binding site of TOP2B as per Figure 2.

previously bound to the γ phosphate of AMPPNP, instead bind to the β phosphate of ADP. The γ phosphate-binding site is now occupied by a sulfate ion present in the crystallization condition (0.2 M ammonium sulfate), as is observed in TOP2A (Wei et al., 2005; Stanger et al., 2014). The sulfate ion is held in place by hydrogen bonds between Y181, G182, and N179, which previously bound the γ phosphate. Thus, our ADP structure represents the post-hydrolysis state with the sulfate group mimicking the ATP hydrolysis product, P_i. The electron density supporting ADP binding is shown in Figure S3B.

Conformational change in the ADP structure to allow release of P_i

Although the GHKL domains of the two structures are virtually identical and the superposition of C-α atoms in this domain gives a root-mean-square deviation (RMSD) value of 0.47 Å, a significant conformational change of the transducer domain occurs during ATP hydrolysis (Figures 4A and 4B). This is represented by an increase in the RMSD value (0.77 Å) when the C-α atoms in this domain are superposed. The transducer domain of the ADP monomer opens up toward the C terminus of the protein (represented by dashed box in 4A), signifying the relaxed conformation. Moreover, during the reorganization of the transducer domain, the QTK loop is shifted away from the ATP-binding site (represented by dashed box in Figures 4B), preventing Q392 and K394 from making contacts with the nucleotide. Specifically, the side chain of Q392 moves by 3.8 Å and K394 moves by 2.5 Å. In turn, this opens the active site for the release of the ATP hydrolysis product P_i.

Comparison of the two human ATPase domain structures, TOP2B and TOP2A

We compared our TOP2B structures with the previously published TOP2A structures (Wei et al., 2005) to understand whether the two

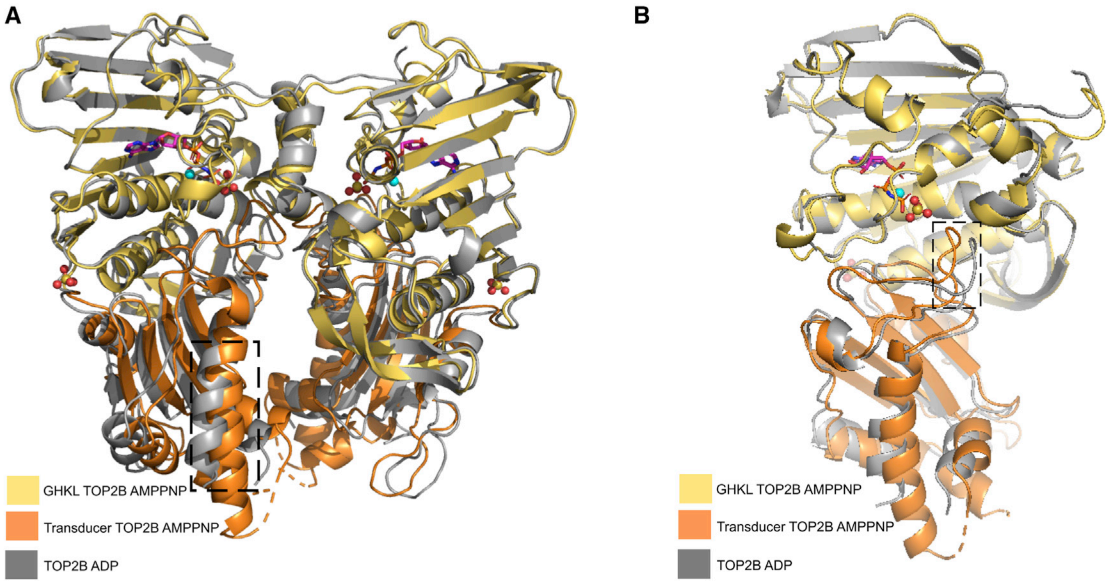

Figure 4. Two alternative views of the superposition of the AMPPNP and ADP overall structures
(A and B) The C-alpha atoms of the GHKL domain of the ADP (gray) structure (residues 45–279) superposed onto the AMPPNP structure colored according to Figure 2 using least-squares superposition. Boxed region highlights the conformational changes that occur during ATP hydrolysis.

human enzymes have similar ATPase domain structures. When bound to AMPPNP, the TOP2A structure is in the restrained conformation and is remarkably similar to TOP2B (Figure 5A). The individual domains possess nearly identical overall structures, with the C-α atoms of the GHKL domain of human TOP2A (28–264) superposing onto TOP2B with an RMSD value of 0.34 Å, while the C-α atoms of the transducer domain of human TOP2A (265–428) superpose onto TOP2B with an RMSD value of 0.69 Å. The nucleotide-binding sites of both structures exhibit a distorted octahedral geometry, with the active-site Mg^{2+} coordinated by the conserved asparagine of TOP2A N91 (N107 in TOP2B), all three phosphates of AMPPNP, and two water molecules, one of which is activated by the catalytic base of TOP2A E87 (E103 in TOP2B).

Both human ADP structures are in an open conformation, suggesting a conformational change is necessary after ATP hydrolysis to accommodate the free phosphate (replaced by a sulfate in both structures) (Figures 2 and 3). As shown in Figure 5B, the transducer domain of our TOP2B structure is in a more open conformation compared with TOP2A, and the C-α atoms of this domain superpose onto TOP2B with an RMSD value of 0.76 Å. Moreover, the β sheets in one of the two GHKL domains do not completely align between human TOP2A and TOP2B (Figure 5B), although the C-α atoms of this domain superpose with an RMSD value of 0.38 Å. This could be due to differences in the internal flexibility of TOP2A and TOP2B or crystallization effects. However, despite these minor differences, overall, the ATPase domains of TOP2A and TOP2B in the presence of either nucleotide are extremely similar.

Comparison of the yeast ATPase domain in complex with ICRF187 reveals similarities in the drug-binding site of TOP2B

To date, the only eukaryotic topoisomerase II ATPase domain structure in the presence of a drug is the *S. cerevisiae* structure in the presence of AMPPNP and dexrazoxane (ICRF187) (Classen et al. 2003). To understand how ICRF187 binds to the clinical target, we co-crystallized the ATPase domain of TOP2B with ICRF187 and collected data; however, there was insufficient electron density to support drug-bound crystals. Therefore, we superposed our TOP2B structure and the TOP2A structure (Wei et al., 2005) onto the *S. cerevisiae* structure bound to ICRF187 to identify the drug-binding site (Figure 6A). Due to the conserved domain organization between type II topoisomerases, the overall structure of the TOP2B ATPase domain is very similar to that of yeast, with an RMSD of value 0.79 Å when using secondary structure matching of all C-α atoms in the protein. The side chains of residues within 3.5 Å of ICRF187 capable of hydrogen bonding are shown in Figure 6B. TOP2A was included in the analysis shown in Figure 6B to determine whether the drug-binding site is comparable between TOP2A and TOP2B. As ICRF187 can inhibit all three enzymes (Roca et al., 1994; Ishida et al., 1995; Hasinoff et al. 2020; Jirkovská et al., 2021; Lee et al. 2017), it was not surprising that the residues were highly conserved in the drug-binding site and all three structures exhibited the same putative 12 residues, six per monomer (H58, T65, Y66, N179, Y181, and Q392 in human TOP2B) involved in hydrogen bonding with ICRF187 (Figure 6B). The side chain with the greatest difference in conformation compared with yeast was the TOP2B residue H58, which is mutated to a tyrosine in two patients (Lam et al. 2017; Hiraide et al., 2020). The equivalent TOP2A residue, H42, also varied in conformation compared with yeast. It is interesting to note that one of the residues found in the drug-binding pocket is Q392, which is involved in binding the γ phosphate of AMPPNP.

Of the six residues directly involved in binding ICRF187, two have been reported in human TOP2A to confer resistance to ICRF187 and ICRF193. Y165S in TOP2A (Y181 in TOP2B) has been described to be resistant to ICRF187 (Wessel et al.,

CellPress
OPEN ACCESS

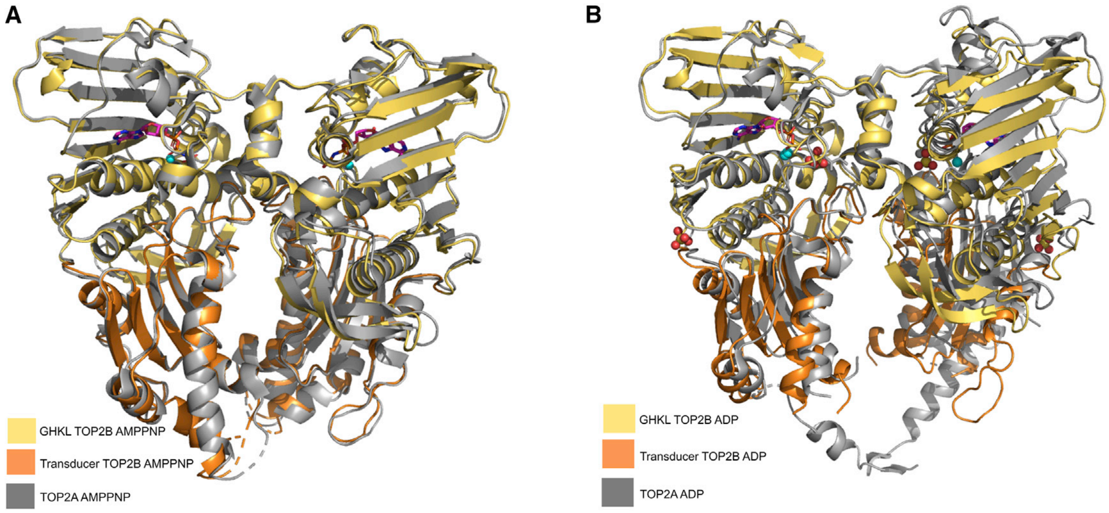

Figure 5. Superposition of the TOP2A and TOP2B structures

(A) The C-alpha atoms of the GHKL domain of human TOP2A (gray) (residues 28–264) in complex with AMPPNP superposed onto the TOP2B AMPPNP structure in Figure 2A using least-squares superposition.

(B) The C-alpha atoms of the GHKL domain of human TOP2A (gray) (residues 28–264) in complex with ADP superposed onto the TOP2B ADP structure in Figure 3A using least-squares superposition.

2002), and Y50F in TOP2A (Y66 in TOP2B) has been shown to confer resistance to ICRF193 (Sehested et al., 1998). Both mutations likely act by disrupting hydrogen bonds between the enzyme and drug, hence reducing drug-binding affinity. In rat TOP2B and human TOP2A, L178F and L169F respectively (L185 in human TOP2B) confer resistance to ICRF187 and ICRF193 (Onoda et al., 2014; Patel et al., 2000). Although L185 in human TOP2B is not directly involved in hydrogen bonding ICRF187, it is near the drug-binding residues N179 and Y181. Similarly, the human TOP2B residue R178Q, which is equivalent to the human TOP2A residue R162Q that confers resistance to ICRF187 (Wessel et al., 1999), does not appear to directly interact with the drug, but mutating it could disrupt the drug-binding pocket.

ICRF193 bound to the clinical target, human TOP2B ATPase domain, in complex with ADP

To define the interactions between the clinical target of the bisdioxopiperazines, we determined the structure of the ATPase domain of human TOP2B in complex with ADP and bound to ICRF193. ICRF193 is a catalytic inhibitor of topoisomerase II that converts the nucleotide-bound ATPase domain to an inactive, closed-clamp intermediate around DNA akin to ICRF187's mechanism of action. ICRF193 is the most potent bisdioxopiperazine, and shows the highest cardioprotective efficiency (Jirkovská et al., 2021), but, due to the low solubility of ICRF193, its use in the clinic has been limited. We report the structure of a type II topoisomerase in complex with ICRF193 (PDB: 7ZBG). Our structure was solved to 2.3 Å by molecular replacement using the ADP-bound structure as a search model. The final TOP2B:ADP:ICRF193 model contains residues 45–422 with a missing loop between residues 354 and 368. The TOP2B:ADP:ICRF193 model was refined to an R_{work} factor of 19.7% and an R_{free} of 25.9% (Table 1). Similar to our previous structures, the TOP2B:ICRF193:ADP-bound structure has one copy of the protein per asymmetric unit and the biological dimer was confirmed by PISA (Krissinel and Henrick 2007). The interface area of the dimer represents ~12% of the total monomer surface, indicating significant interactions, which include 36 hydrogen bonds and 12 salt bridges between the two monomers.

The overall structure of the ADP:ICRF193 model is virtually unchanged compared with the ADP structure without drug (Figure 7A). The nucleotide-binding sites are extremely similar in the presence and absence of ICRF193 and superpose with an RMSD value of 0.19 Å. Moreover, the transducer domains superpose with near identity and exhibit an RMSD value of 0.35 Å. A single drug molecule was observed bound in the previously reported ICRF187-binding site (Classen et al. 2003), bound in one of two possible conformations (Figure 7B). The same protein drug interactions are observed in either binding mode due to the pseudo-symmetry of ICRF193, which stabilizes the nucleotide-bound transient dimer interface between two ATPase protomers. The drug-binding pocket consists of the same 12 amino acids (six from each protomer) involved in hydrogen bonding ICRF187. The GHKL domain contributes the majority of residues (H58, T65, Y66, N179, and Y181), with only one transducer domain residue, Q392, binding to the drug. Y66 makes direct contact with ICRF193, accounting for the resistant phenotype when the equivalent residue in human TOP2A (Y50) is mutated to a phenylalanine (Sehested et al., 1998). Similar to the ICRF187 *S. cerevisiae*-binding site, superpositions of the drug-free and ICRF193-bound structures show that the drug-binding site is a preformed feature of the nucleotide-bound dimerized protein. However, the side chain of Q392 moves by ~4.5 Å in order to accommodate ICRF193 and prevent a steric clash occurring between C9 of ICRF193 and the amide nitrogen of Q392 (Figure 7C). The movement of Q392 to accommodate drug has not been previously reported as the equivalent yeast residue (Q365) is in the same conformation in the presence and absence

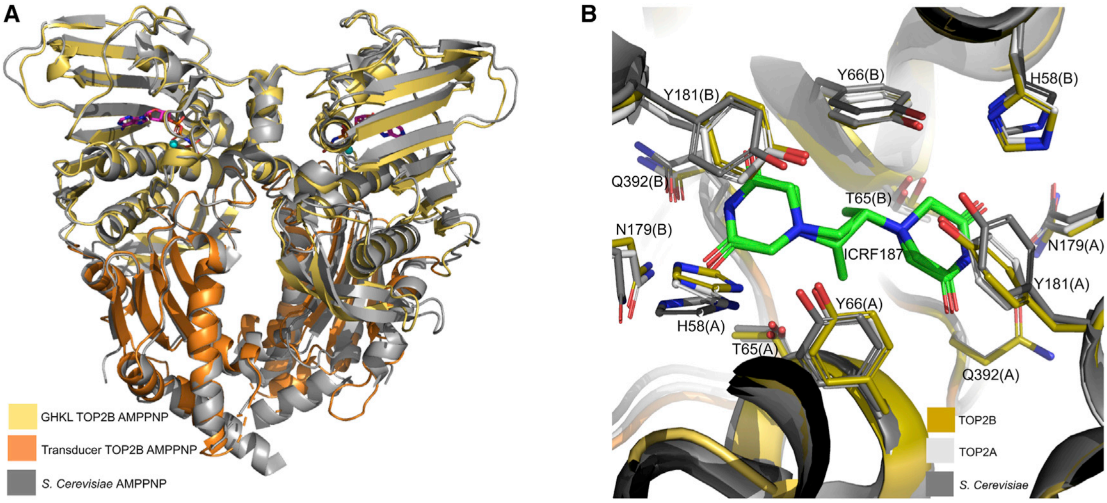

Figure 6. Superposition of the *S. cerevisiae* TOP2 ATPase domain structure onto TOP2B AMPPNP structure and drug-binding site

(A) The C-alpha atoms of the *S. cerevisiae* ATPase domain (gray) (residues 7–243) in complex with AMPPNP and ICRF187 superposed onto the human TOP2B structure in complex with AMPPNP colored according to Figure 2A using least-squares superposition. ICRF187 is colored by atom: carbon, green; oxygen, red; and nitrogen, blue.

(B) The ICRF187-binding site of *S. cerevisiae* (dark gray) and TOP2A (silver) superposed onto the TOP2B ATPase structure colored according to Figure 2A. The side chains of residues within 3.5 Å of ICRF187 are shown in stick representation and colored by atom. Residue labels according to human TOP2B numbering. (A)/(B) denotes the protein chain.

of ICRF187 when bound to AMPPNP (Classen et al. 2003). During ATP hydrolysis, Q392 moves toward the drug-binding site as represented in the ADP-bound structure (Figure 4), thus a further conformational change of Q392 is required in order to accommodate ICRF193 in the presence of ADP, which is not necessary when bound to AMPPNP. An alternative rotamer of T65 is observed to allow a hydrogen bond to form to the hydroxyl group of ICRF193. ICRF193 is similar in structure to ICRF187 but contains an additional methyl group on the ethanediyl linker, hence the bisdioxopiperazine-binding site is large enough to accommodate either form of the drug. However, the presence of one more methyl group allows ICRF193 to have an additional contact with Y66, which perhaps accounts for the increased potency of the drug.

ATPase activity of human TOP2B proteins reveals that the presence of the N-terminal strap reduces the rate of ATP hydrolysis

All type II topoisomerases contain an N-terminal strap that forms part of the ATP-binding site. *S. cerevisiae* has the shortest N-terminal strap (Figure 1B), and, in the *S. cerevisiae* crystal structures, the beginning of the N-terminal strap is absent from the models as there is no electron density for the first six residues. The human type II topoisomerases have longer N-terminal strap regions than the *S. cerevisiae* TOP2 (Figure 1B), and previous proteolysis and N-terminal sequencing revealed proteolytic sensitive sites at E47 and R48 in TOP2B and E31 in TOP2A (Austin et al., 1995), indicating this region of the N-terminal strap is accessible. The TOP2B proteins crystallized here and those reported for TOP2A (Wei et al., 2005) do not contain the full N-terminal strap. Hence, there are no eukaryotic TOP2 ATPase domain structures containing the complete N-terminal strap, which is the most variable region of the ATPase domain in both amino acid sequence and post-translational modification sites. We attempted crystallizing the full-length ATPase (1–444), but we were unable to obtain crystals. Therefore, to gain a more in-depth understanding of the function of the N-terminal strap, we biochemically characterized the complete human TOP2B ATPase domain (1–444) as well as the alternative splice variant (1–449) and the recombinant ATPase domain (residues 45–444) that was expressed and purified for crystallography to ensure the protein was functional. Furthermore, E103 was selected for mutagenesis due to its likely function as the catalytic base (Figures 2C and 2D). E103 was mutated to an alanine in both vectors (45–444 and 1–444) and ATP hydrolysis was measured. The ability of TOP2B proteins to carry out ATP hydrolysis was assayed spectrophotometrically by measuring the release of free phosphate via a color change. Unless otherwise stated, 1 μM protein was added to the reaction mixture containing 0.1 mM ATP.

In the presence of 0.1 mM ATP, TOP2B (45–444) had a significantly higher rate of ATP hydrolysis compared with the full-length ATPase domain (residues 1–444; $p < 0.001$) and the alternative splice variant (residues 1–449; $p < 0.001$) TOP2B proteins (Figure 8B). TOP2B 1–444 had 36% ATPase activity at 1 μM compared with TOP2B 45–444, while TOP2B 1–449 was marginally more active with 46% ATPase activity at 1 μM compared with TOP2B 45–444. However, this increase in activity between 1 and 444 and 1 and 449 did not reach significance ($p > 0.05$). The E103A mutation in both the 45–444 and 1–444 vector could not catalyze ATP hydrolysis under any of the conditions tested. This confirms that the glutamate at position 103 is essential for ATP hydrolysis and any observed ATPase activity in the three wild-type proteins is a result of functional TOP2B protein.

ATPase activity was also assayed at a range of ATP concentrations (0.05–0.5 mM) (Figure 8C). As expected, increasing the

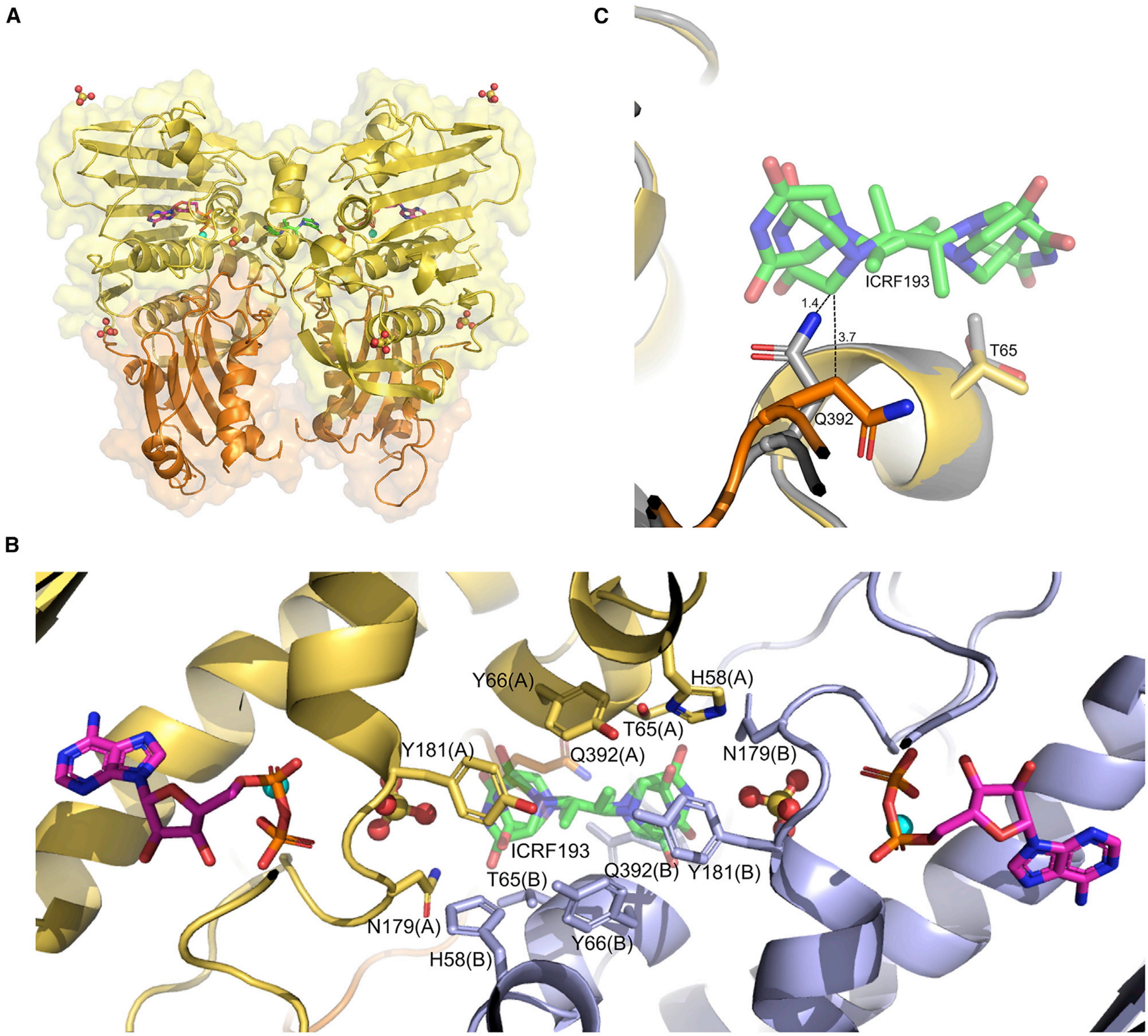

Figure 7. Overall structure and binding sites of the ATPase domain of human TOP2B bound to ADP:ICRF193

(A) The GHKL and transducer domains of TOP2B are colored in yellow and orange respectively. ADP colored by atom: carbon, pink; nitrogen, blue; oxygen, red; and phosphate, orange. The surface representation is also shown. Mg^{2+} ions colored in cyan. SO_4 is represented by oxygen in red spheres and sulfur in yellow spheres. ICRF193 binds between the two TOP2B protomers and is colored by atom: carbon, green; nitrogen, blue; and oxygen, red.

(B) The side chains of residues involved in binding to ICRF193 are shown in stick representation. GHKL and transducer residues from one monomer are colored in yellow and orange respectively, and the other monomer colored in pale blue. (A)/(B) denotes protein chain. Both possible binding conformations of ICRF193 are shown.

(C) The C-alpha atoms of the ADP complex in the absence of drug (gray) superposed onto the ADP:ICRF193 structure (yellow and orange) using least-squares superposition. Q392 moves away from drug-binding site in the ADP:ICRF193 structure to create space for the drug. Dashed lines represent the distance in angstroms. Without the conformational change, Q392 is 1.4 Å from the C9 of ICRF193, whereas, upon repositioning the side chain, there is now a distance of 3.7 Å. Alternative conformer of T65 for formation of an extra hydrogen bond to ICRF193. Other side chains not shown as they have the same conformation in the presence and absence of ICRF193 bound to ADP.

ATP concentration increased the amount of free phosphate released for all TOP2B proteins. However, the ATPase activity of TOP2B (45–444) began to reach a maximum at ATP concentrations of 0.4 mM and above. By contrast, the ATPase activity of the full-length TOP2B ATPase domain (1–444 and 1–449) increased linearly at all ATP concentrations studied.

Upon addition of pBR322 (plasmid DNA), all three proteins' ATPase activity at 1 μM protein concentration was significantly stimulated ($p < 0.001$ for all cases) (Figure 8D). TOP2B 1–444 had the largest percentage increase (72%) in activity upon addition of DNA, followed by TOP2B 1–449 (61%), while TOP2B 45–444 had the smallest increase (53%) in ATPase

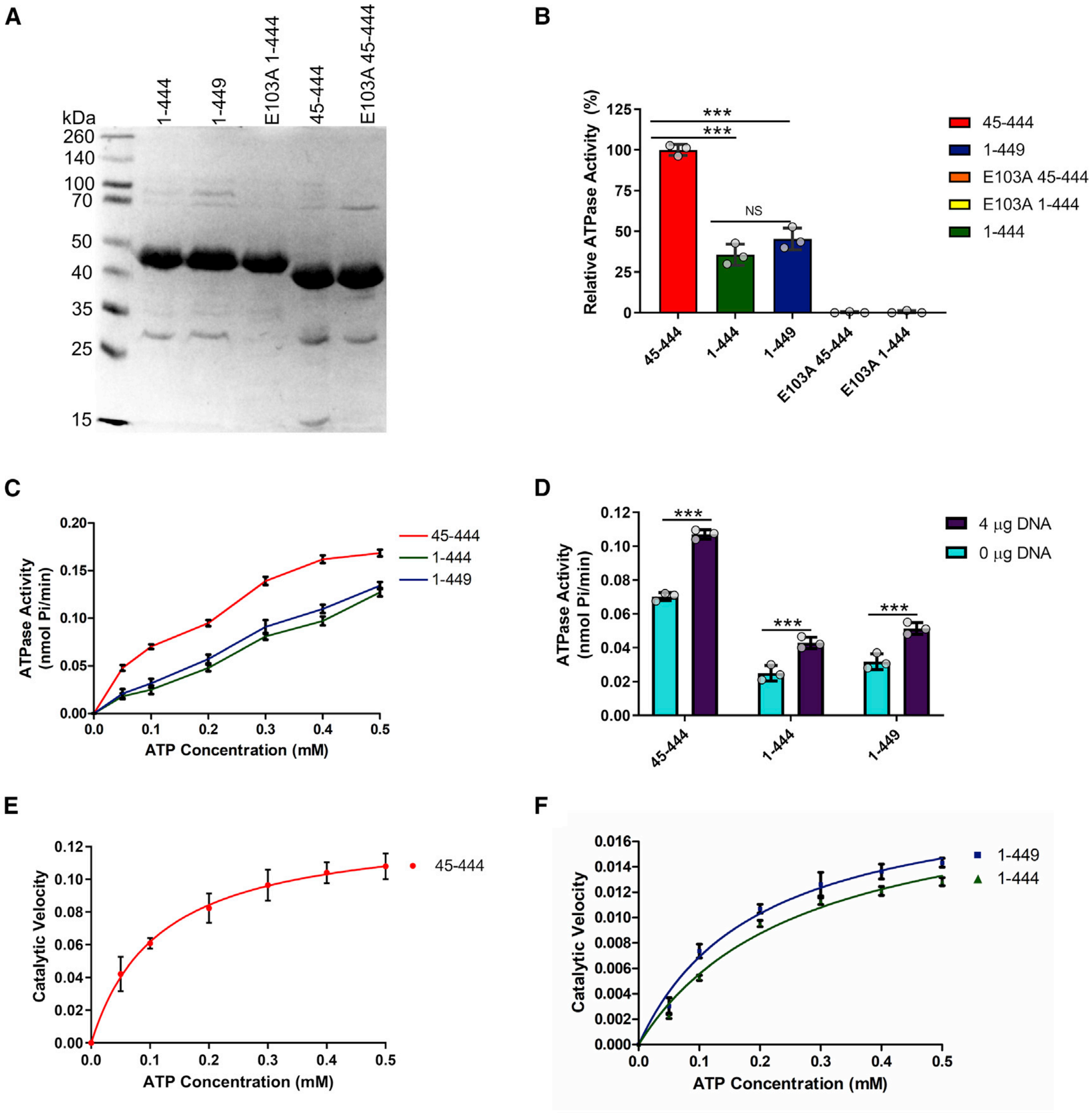

G

Protein	K_m Value	V_{max} Value
45-444	0.1153 +/- 0.0067	0.1328 +/- 0.0025
1-444	0.2670 +/- 0.0697	0.0204 +/- 0.0025
1-449	0.1957 +/- 0.0397	0.0204 +/- 0.0017

+/- Standard error

Figure 8. ATP hydrolysis by topoisomerase II ATPase domain proteins

(A) Ten percent SDS polyacrylamide gel with the purified human topoisomerase II ATPase domain proteins. Each lane contains 15 µL of sample and 5 µL of SDS loading buffer. Lane 1, molecular markers, lane 2, TOP2B 1-444; lane 3, TOP2B 1-449; lane 4, TOP2B 1-444 E103A; lane 5, TOP2B 45-444; lane 6, TOP2B 45-444 E103A.

(B) Comparison of relative ATP hydrolysis activities for the different ATPase domain proteins. The mean for TOP2B 45–444 was set to 100%. The different proteins were assayed for activity at a single concentration of 1 µM, the means of three replicates are shown as histograms, and the individual data points (n = 3) are shown as open circles on the histogram. Error bars represent one standard deviation from the mean. Red is TOP2B 45–444, green is TOP2B 1–444, and blue is TOP2B 1–449. Statistical analysis was performed by a one-way ANOVA and Tukey's multiple comparison test. ***$p < 0.001$; NS, non-significant.

(C) ATP hydrolysis by topoisomerase II ATPase domain proteins at a range of ATP concentrations. ATPase activity indicated by the amount of free phosphate released per minute for each of the ATPase domain proteins at a range of ATP concentrations (0.05, 0.1, 0.2, 0.3, 0.4, and 0.5 mM ATP). The different proteins were assayed for activity at a concentration of 1 µM and the means of three replicates are shown. Error bars represent one standard deviation from the mean. Same color scheme used as in (B).

(D) ATP hydrolysis by topoisomerase II ATPase domain proteins in the presence and absence of DNA at 1 µM protein concentration. The different proteins were assayed for activity in the presence and absence of 4 µg of pBR322 at a single concentration of 1 µM, the means of three replicates are shown as histograms, and the individual data points (n = 3) are shown as open circles on the histogram. Error bars represent one standard deviation from the mean. Activity in the absence of

(legend continued on next page)

activity when DNA was added. Moreover, the ATPase activity of TOP2B (45–444) at 2 and 2.5 μM protein concentrations in the presence and absence of DNA was not significantly different, indicating it had reached a maximum. However, at 2.5 μM protein concentration, the ATPase activity of the two full-length proteins (1–444 and 1–449) was still significantly stimulated in the presence of DNA (data not shown).

The ATPase activity of the TOP2B proteins at 0.25 μM was assayed over time at a range of ATP concentrations and the data were fitted to the Michaelis-Menten equation, as illustrated by the best-fit curves in Figures 8E and 8F (solid curve). The K_m value for TOP2B 45–444 (0.1150 mM) was significantly lower than the value obtained for TOP2B 1–444 (0.2670 mM) and TOP2B 1–449 (0.1957 mM) shown in Figure 8G. TOP2B 45–444 has a higher affinity for the substrate (ATP) compared with TOP2B 1–444 and TOP2B 1–449. There was no significant difference in the K_m values for TOP2B 1–444 and TOP2B 1–449. Moreover, the V_{max} value was the same for both TOP2B 1–444 and TOP2B 1–449 at 0.0204 nmol/min, whereas TOP2B 45–444 had a significantly higher value at 0.1328 nmol/min (note the different scales in E and F). As such, TOP2B 45–444 is more active compared with TOP2B 1–444 and TOP2B 1–449.

DISCUSSION

Our data show that the ATPase domain of human TOP2B in complex with AMPPNP is in the restrained conformation with the nucleotide-binding site sequestered from solvent (Figure 2). The cavity between the two monomers is not large enough to easily accommodate a DNA duplex. Therefore, upon T-DNA binding, strain is induced within the ATPase dimer, which is hypothesized to promote DNA cleavage of the G-DNA. By contrast, our ADP structure is in a more open, relaxed confirmation (Figure 3), with the QTK loop from the transducer domain undergoing a distinct movement away from the nucleotide-binding site (Figure 4). This movement then accommodates a sulfate group, in place of the ATP hydrolysis product, P_i (Figures 3C and 3D). Remarkably, our structures demonstrate that ATP hydrolysis results in a conformational change of the transducer domain, while the GHKL domain remains extremely similar between the two nucleotide-bound states (Figure 4A). The catalytic Mg^{2+} ion is coordinated by a conserved asparagine residue (N107), all three phosphates of AMPPNP, and two water molecules, one of which becomes activated by the catalytic glutamate residue (E103). As expected, when this residue is mutated to an alanine, ATP hydrolysis is abolished (Figure 8B).

The bisdioxopiperazine dexrazoxane (ICRF187) is approved for use in the clinic to reduce the incidence of anthracycline-induced heart failure (van Dalen et al., 2011; Bansal et al., 2021). The cardioprotective effect of ICRF187 acts via inhibiting or depleting the TOP2 isoform in cardiomyocytes, TOP2B. Although we were unsuccessful in obtaining an ICRF187 TOP2B-bound structure, we obtained a structure of the ATPase domain of human TOP2B bound to ADP:ICRF193, experimentally demonstrating that ICRF193 binds within the same binding site as was reported for *S. cerevisiae* TOP2 and ICRF187 (Figures 6 and 7). Two main conformational changes occur in order to accommodate ICRF193 within the binding site. The side chain of Q392 from the QTK loop moves by ~4.5 Å to prevent a steric clash with the drug, thus it is likely that mutating position Q392 to a larger residue would result in resistance to ICRF193 as the drug-binding site would be restricted. Second, an alternative rotamer of T65 occurs to form an additional hydrogen bond to ICRF193. ICRF193 displays a higher potency against TOP2B compared with ICRF187(Hasinoff et al., 1995; Hasinoff et al. 2020; Jirkovská et al., 2021), likely due to the extra methyl group in ICRF193, which forms an additional hydrogen bond to Y66.

Our TOP2B structures share an overall tertiary structure and transducer opening mechanism very similar to TOP2A (Figure 5). In both human structures and the *S. cerevisiae* structure, the beginning of the N-terminal strap is absent. Hence, there are no eukaryotic TOP2 ATPase domain structures containing the complete N-terminal strap, which is the most variable region of the ATPase domain in both amino acid sequence and post-translational modification sites. *S. cerevisiae* has the shortest N-terminal strap, followed by human TOP2A and then human TOP2B. Moreover, human TOP2B has the most phosphorylation sites within this region, which include S4, T21, A32, S37, S45, and S46; by contrast, human TOP2A is only phosphorylated at S4 and S29 (Hornbeck et al., 2014). In addition, TOP2B can be SUMOylated at K28 and K29 in the N-terminal strap (Hendriks et al., 2014). As the amino acids in the N-terminal strap are not conserved between isoforms or species, the presence of the strap could provide a mechanism of enzyme regulation modulated by post-translational modifications. Indeed, it is known that the N-terminal strap has a functional role both *in vitro* and *in vivo* as we have determined that the full-length TOP2B protein (1–444) and the alternative splice variant (1–449) are less active than the N-terminal truncated protein (45–444) (Figure 8). One explanation for this could be that the additional amino acids in the N-terminal strap sequester ADP and P_i and prevent them from leaving the enzyme as easily. As such, it would take longer to reset the enzyme for another round of catalysis, resulting in a slower ATP hydrolysis rate. *In vivo*, it has been demonstrated that the N-terminal strap is necessary for interacting with PKC in *S. cerevisiae* TOP2 (Mouchel and Jenkins 2006) and the phosphorylation status of the strap can modulate activity in human TOP2A (Wells and Fry 1995). It has previously been proposed that the phosphorylation status of a different region of TOP2, the C-terminal domain, can alter the conformation of the protein, enabling interactions with other molecules or proteins (Cardenas

nucleic acid is shown in cyan and activity in the presence of 4 μg of nucleic acid shown in purple. Statistical analysis was performed by a two-way ANOVA and Bonferroni post-tests. ***$p < 0.001$.

(E) Michaelis-Menten graph for TOP2B 45–444. Rate of ATP hydrolysis plotted against ATP concentration for 0.25 μM protein. Error bars represent one standard deviation from the mean (n = 3).

(F) Michaelis-Menten graph for TOP2B 1–444 and TOP2B 1–449 at 0.25 μM protein. Note different scale to (E). Error bars represent one standard deviation from the mean (n = 3).

(G) Table with K_m and V_{max} values ± standard error for TOP2B 45–444, TOP2B 1–444, and TOP2B 1–449.

and Gasser 1993). Hence, a similar event could occur with the N-terminal strap whereby phosphorylation can promote interaction with ATP or interfere with the accessibility of the ATP-binding site, which could account for the difference in activity between the full-length (1–444 and 1–449) and the N-terminal truncated proteins (45–444).

Type II topoisomerase enzymes are multidomain proteins and couple a DNA cleavage and religation reaction to two independent ATP hydrolysis events in order to regulate DNA topology. During this reaction, two DNA duplexes are captured. Once both duplexes are bound, passage of the T-DNA through the break in the G-DNA requires the energy generated by ATP hydrolysis. It has previously been shown that TOP2B (45–1621) ATPase activity is stimulated by DNA (West et al., 2002); consistent with this, here we show that the ATPase activity of the ATPase domains is stimulated by DNA. Similar results have been reported for the TOP2A ATPase domain, yeast ATPase domain, and gyrase B ATPase domain (Campbell and Maxwell 2002; Schmidt et al. 2012; Maxwell and Gellert 1984). The DNA stimulation of the ATPase activity was presumed to be due to binding of the T-DNA; however, in addition to the T-DNA, the K loop in the transducer region of the ATPase domain also contacts the G-DNA, and mutagenesis of the K loop in yeast TOP2 and human TOP2A confirmed that this region of the ATPase domain is responsible for DNA stimulation of ATP hydrolysis (Schmidt et al. 2012). Once the G-DNA has been religated, it contacts the K loop to stimulate the second ATP hydrolysis event to reset the enzyme for another round of catalysis. One of the lysine residues in the K loop of *S. cerevisiae* was previously implicated in binding DNA, as demonstrated from a protein footprinting study (Li and Wang 1997). Also, lysine residues in the K loop of TOP2B have been found to be acetylated, adding a further potential layer of regulation (Schmidt et al. 2012). As the K loop resides within the transducer region of the ATPase domain, the transducer has been implicated as an essential element for this allosteric movement (Vanden Broeck et al., 2021). By coupling DNA binding to ATPase stimulation, the enzyme has a regulatory mechanism that prevents ATP from being hydrolyzed unnecessarily when there is no DNA substrate in proximity.

We report the crystal structure for the ATPase domain of human TOP2B, in complex with AMPPNP or ADP, and in the presence of ICRF193. These crystal structures provide a valuable resource to study drug interactions with the ATPase domain. The biochemical analysis of the ATPase domain with the full N-terminal strap indicates that the first 44 or 49 amino acids can negatively regulate the ATPase activity, providing a possible further means to regulate TOP2B activity.

STAR★METHODS

Detailed methods are provided in the online version of this paper and include the following:

- KEY RESOURCES TABLE
- RESOURCE AVAILABILITY
  - Lead contact
  - Materials availability
  - Data and code availability
- EXPERIMENTAL MODEL AND SUBJECT DETAILS
- METHOD DETAILS
  - Cloning
  - Expression and purification
  - Crystallization
  - Data collection and processing
  - Phase problem and model building
  - ATPase activity assays
- QUANTIFICATION AND STATISTICAL ANALYSIS

SUPPLEMENTAL INFORMATION

Supplemental information can be found online at https://doi.org/10.1016/j.str.2022.05.009.

ACKNOWLEDGMENTS

The authors would like to thank Diamond Light Source for beamtime (proposal mx24948), and the staff of beamline I03 and I24. This work was supported by a Biotechnology and Biological Sciences Research Council DTP2 Training Grant (project reference: BB/M011186/1) to E.M.L.; Newcastle University support to I.G.C., A.B., B.v.d.B, and C.A.A.; and Durham University support to T.R.B.

AUTHOR CONTRIBUTIONS

Conceptualization, E.M.L., C.A.A., and T.R.B.; methodology, E.M.L., A.B., C.A.A., T.R.B., and B.v.d.B.; investigation, E.M.L. and A.B.; writing – original draft, E.M.L., C.A.A., T.R.B., and I.G.C.; writing – review & editing, E.M.L., C.A.A., I.G.C., T.R.B., A.B., and B.v.d.B.; funding acquisition, C.A.A.; resources, C.A.A., A.B., I.G.C., T.R.B., and B.v.d.B.; supervision, C.A.A., T.R.B., B.v.d.B., I.G.C., and A.B.

DECLARATION OF INTERESTS

The authors declare no competing interests.

Received: January 21, 2022
Revised: March 25, 2022
Accepted: May 10, 2022
Published: June 3, 2022

REFERENCES

Aguet, F., Shankara, A., Ardlie, K.G., Stacey, G., Getz, G.A., Aaron, G., Kane, H., Handsaker, R.E., Huang, K.H., Seva, K., et al. (2020). The GTEx Consortium atlas of genetic regulatory effects across human tissues. Science *369*, 1318–1330.

Ali, J.A., Jackson, A.P., Howells, A.J., and Maxwell, A. (1993). The 43-kilodalton N-terminal fragment of the DNA gyrase B protein hydrolyzes ATP and binds coumarin drugs. Biochemistry *32*, 2717–2724.

Atwal, M., Swan, R.L., Rowe, C., Lee, K.C., Lee, D.C., Armstrong, L., Cowell, I.G., and Austin, C.A. (2019). Intercalating TOP2 poisons attenuate topoisomerase action at higher concentrations. Mol. Pharmacol. *96*, 475–484.

Austin, C.A., Cowell, I.G., Khazeem, M.M., Lok, D., and Ng, H.T. (2021). TOP2B's contributions to transcription. Biochem. Soc. Trans. *49*, 2483–2493.

Austin, C.A., Marsh, K.L., Wasserman, R.A., Willmore, E., Sayer, P.J., Wang, J.C., and Fisher, L.M. (1995). Expression, domain structure, and enzymatic properties of an active recombinant human DNA topoisomerase II beta. J. Biol. Chem. *270*, 15739–15746.

Austin, C.A., Sng, J.H., Patel, S., and Fisher, L.M. (1993). Novel HeLa topoisomerase II is the II beta isoform: complete coding sequence and homology with other type II topoisomerases. Biochim. Biophys. Acta *1172*, 283–291.

Baird, C.L., Gordon, M.S., Andrenyak, D.M., Marecek, J.F., and Lindsley, J.E. (2001). The ATPase reaction cycle of yeast DNA topoisomerase II. Slow rates of ATP resynthesis and P(i) release. J. Biol. Chem. *276*, 27893–27898.

CellPress
OPEN ACCESS

Baird, C.L., Harkins, T.T., Morris, S.K., and Lindsley, J.E. (1999). Topoisomerase II drives DNA transport by hydrolyzing one ATP. Proc. Natl. Acad. Sci. U S A. *96*, 13685–13690.

Bansal, N., Joshi, C., Adams, M.J., Hutchins, K., Ray, A., and Lipshultz, S.E. (2021). Cardiotoxicity in pediatric lymphoma survivors. Expet. Rev. Cardiovasc. Ther. *19*, 957–974.

Bedez, C., Lotz, C., Batisse, C., Vanden Broeck, A., Stote, R.H., Howard, E., Pradeau-Aubreton, K., Ruff, M., and Lamour, V. (2018). Post-translational modifications in DNA topoisomerase 2α highlight the role of a eukaryote-specific residue in the ATPase domain. Sci. Rep. *8*, 9272.

Berger, J.M., Gamblin, S.J., Harrison, S.C., and Wang, J.C. (1996). Structure and mechanism of DNA topoisomerase II. Nature *379*, 225–232.

Bergerat, A., de Massy, B., Gadelle, D., Varoutas, P.C., Nicolas, A., and Forterre, P. (1997). An atypical topoisomerase II from Archaea with implications for meiotic recombination. Nature *386*, 414–417.

Brino, L., Urzhumtsev, A., Mousli, M., Bronner, C., Mitschler, A., Oudet, P., and Moras, D. (2000). Dimerization of Escherichia coli DNA-gyrase B provides a structural mechanism for activating the ATPase catalytic center. J. Biol. Chem. *275*, 9468–9475.

Broderick, L., Yost, S., Dong, L., McGeough, M.D., Booshehri, L.M., Guaderrama, M., Brydges, S.D., Kucharova, K., Patel, N.C., Harr, M., et al. (2019). Mutations in topoisomerase IIβ result in a B cell immunodeficiency. Nat. Commun. *10*, 3644.

Campbell, S., and Maxwell, A. (2002). The ATP-operated clamp of human DNA topoisomerase IIalpha: hyperstimulation of ATPase by "piggy-back" binding. J. Mol. Biol. *320*, 171–188.

Capranico, G., Tinelli, S., Austin, C.A., Fisher, M.L., and Zunino, F. (1992). Different patterns of gene expression of topoisomerase II isoforms in differentiated tissues during murine development. Biochim. Biophys. Acta *1132*, 43–48.

Cardenas, M.E., and Gasser, S.M. (1993). Regulation of topoisomerase II by phosphorylation: a role for casein kinase II. J. Cell Sci. *104* (Pt 2), 219–225.

Classen, S., Olland, S., and Berger, J.M. (2003). Structure of the topoisomerase II ATPase region and its mechanism of inhibition by the chemotherapeutic agent ICRF-187. Proc. Natl. Acad. Sci. U S A. *100*, 10629–10634.

Corbett, K.D., and Berger, J.M. (2003). Structure of the topoisomerase VI-B subunit: implications for type II topoisomerase mechanism and evolution. Embo J *22*, 151–163.

Corbett, K.D., and Berger, J.M. (2004). Structure, molecular mechanisms, and evolutionary relationships in DNA topoisomerases. Annu. Rev. Biophys. Biomol. Struct. *33*, 95–118.

Corbett, K.D., Schoeffler, A.J., Thomsen, N.D., and Berger, J.M. (2005). The structural basis for substrate specificity in DNA topoisomerase IV. J. Mol. Biol. *351*, 545–561.

Davies, S.L., Jenkins, J.R., and Hickson, I.D. (1993). Human cells express two differentially spliced forms of topoisomerase II beta mRNA. Nucleic. Acids. Res. *21*, 3719–3723.

Deng, S., Yan, T., Jendrny, C., Nemecek, A., Vincetic, M., Gödtel-Armbrust, U., and Wojnowski, L. (2014). Dexrazoxane may prevent doxorubicin-induced DNA damage via depleting both topoisomerase II isoforms. BMC. Cancer. *14*, 842.

Dutta, R., and Inouye, M. (2000). GHKL, an emergent ATPase/kinase superfamily. Trends Biochem. Sci. *25*, 24–28.

Emsley, P., Lohkamp, B., Scott, W.G., and Cowtan, K. (2010). Features and development of coot. Acta. Crystallogr. Sect. D Biol. Crystallogr. *66*, 486–501.

Evans, P.R., and Murshudov, G.N. (2013). How good are my data and what is the resolution? Acta. Crystallogr. D. *69*, 1204–1214.

Gardiner, L.P., Roper, D.I., Hammonds, T.R., and Maxwell, A. (1998). The N-terminal domain of human topoisomerase IIalpha is a DNA-dependent ATPase. Biochemistry *37*, 16997–17004.

Gilroy, K.L., Leontiou, C., Padget, K., Lakey, J.H., and Austin, C.A. (2006). mAMSA resistant human topoisomerase IIbeta mutation G465D has reduced ATP hydrolysis activity. Nucleic Acids Res. *34*, 1597–1607.

Hammonds, T.R., and Maxwell, A. (1997). The DNA dependence of the ATPase activity of human DNA topoisomerase IIalpha. J. Biol. Chem. *272*, 32696–32703.

Harkins, T.T., Lewis, T.J., and Lindsley, J.E. (1998). Pre-steady-state analysis of ATP hydrolysis by Saccharomyces cerevisiae DNA topoisomerase II. 2. Kinetic mechanism for the sequential hydrolysis of two ATP. Biochemistry *37*, 7299–7312.

Hasinoff, B.B., Kuschak, T.I., Yalowich, J.C., and Creighton, A.M. (1995). A QSAR study comparing the cytotoxicity and DNA topoisomerase II inhibitory effects of bisdioxopiperazine analogs of ICRF-187 (dexrazoxane). Biochem. Pharmacol. *50*, 953–958.

Hasinoff, B.B., Patel, D., and Wu, X. (2020). A QSAR study that compares the ability of bisdioxopiperazine analogs of the doxorubicin cardioprotective agent dexrazoxane (ICRF-187) to protect myocytes with DNA topoisomerase II inhibition. Toxicol. Appl. Pharmacol. *399*, 115038.

Hearnshaw, S.J., Chung, T.T.-H., Stevenson, C.E.M., Maxwell, A., and Lawson, D.M. (2015). The role of monovalent cations in the ATPase reaction of DNA gyrase. Acta Crystallogr. Sect. D Biol. Crystallogr. *71*, 996–1005.

Hendriks, I.A., D'Souza, R.C.J., Yang, B., Verlaan-de Vries, M., Mann, M., Alfred, C., and Vertegaal, O. (2014). Uncovering global SUMOylation signaling networks in a site-specific manner. Nat. Struct. Mol. Biol. *21*, 927–936.

Hiraide, T., Watanabe, S., Matsubayashi, T., Yanagi, K., Nakashima, M., Ogata, T., and Saitsu, H. (2020). A de novo TOP2B variant associated with global developmental delay and autism spectrum disorder. Mol. Genet. Genomic. Med. *8*, e1145.

Hornbeck, P.V., Zhang, B., Murray, B., Kornhauser, J.M., Latham, V., and Skrzypek, E. (2014). PhosphoSitePlus mutations, PTMs and recalibrations. Nucleic. Acids. Res. *43*, 512–520.

Ishida, R., Hamatake, M., Wasserman, R.A., Nitiss, J.L., Wang, J.C., and Andoh, T. (1995). DNA topoisomerase II is the molecular target of bisdioxopiperazine derivatives ICRF-159 and ICRF-193 in Saccharomyces cerevisiae. Cancer. Res. *55*, 2299–2303.

Jirkovská, A., Karabanovich, G., Kubeš, J., Skalická, V., Melnikova, I., Korábečný, J., Kučera, T., Jirkovský, E., Nováková, L., Bavlovič Piskáčková, H., et al. (2021). Structure-activity relationship study of dexrazoxane analogues reveals ICRF-193 as the most potent bisdioxopiperazine against anthracycline toxicity to cardiomyocytes due to its strong topoisomerase IIβ interactions. J. Med. Chem. *64*, 3997–4019.

Krissinel, E., and Henrick, K. (2007). Inference of macromolecular assemblies from crystalline state. J. Mol. Biol. *372*, 774–797.

Lam, C.W., Yeung, W.L., and Law, C.Y. (2017). Global developmental delay and intellectual disability associated with a de novo TOP2B mutation. Clin. Chim. Acta *469*, 63–68.

Lamour, V., Hoermann, L., Jeltsch, J.M., Oudet, P., and Moras, D. (2002). An open conformation of the Thermus thermophilus gyrase B ATP-binding domain. J. Biol. Chem. *277*, 18947–18953.

Lee, J.H., Wendorff, T.J., and Berger, J.M. (2017). Resveratrol: a novel type of topoisomerase II inhibitor. J. Biol. Chem. *292*, 21011–21022.

Li, W., and Wang, J.C. (1997). Footprinting of yeast DNA topoisomerase II lysyl side chains involved in substrate binding and interdomainal interactions. J. Biol. Chem. *272*, 31190–31195.

Lindsley, J.E., and Wang, J.C. (1993). On the coupling between ATP usage and DNA transport by yeast DNA topoisomerase II. J. Biol. Chem. *268*, 8096–8104.

Lyu, Y.L., and Wang, J.C. (2003). 'Aberrant lamination in the cerebral cortex of mouse embryos lacking DNA topoisomerase IIbeta. Proc. Natl. Acad. Sci. U S A. *100*, 7123–7128.

Maxwell, A., and Gellert, M. (1984). 'The DNA dependence of the ATPase activity of DNA gyrase. J. Biol. Chem. *259*, 14472–14480.

McCoy, A.J., Grosse-Kunstleve, R.W., Adams, P.D., Winn, M.D., Storoni, L.C., and Read, R.J. (2007). Phaser crystallographic software. J. Appl. Crystallogr. *40*, 658–674.

Meczes, E.L., Marsh, K.L., Fisher, L.M., Rogers, M.P., and Austin, C.A. (1997). 'Complementation of temperature-sensitive topoisomerase II mutations in Saccharomyces cerevisiae by a human TOP2 beta construct allows the study of topoisomerase II beta inhibitors in yeast. Cancer Chemother. Pharmacol. *39*, 367–375.

Moreira, F., Arenas, M., Videira, A., and Pereira, F. (2022). 'Evolutionary history of TOPIIA topoisomerases in animals. J. Mol. Evol. *90*, 149–165.

Morris, S.K., Baird, C.L., and Lindsley, J.E. (2000). Steady-state and rapid kinetic analysis of topoisomerase II trapped as the closed-clamp intermediate by ICRF-193 *. J. Biol. Chem. *275*, 2613–2618.

Mouchel, N.A.P., and Jenkins, J.R. (2006). The identification of a functional interaction between PKC and topoisomerase II. FEBS (Fed. Eur. Biochem. Soc.) Lett. *580*, 51–57.

Murshudov, G.N., Skubak, P., Lebedev, A.A., Pannu, N.S., Steiner, R.A., Nicholls, R.A., Winn, M.D., Long, F., and Vagin, A.A. (2011). REFMAC5 for the refinement of macromolecular crystal structures. Acta. Crystallogr. D. *67*, 355–367.

Oestergaard, V.H., Bjergbaek, L., Skouboe, C., Giangiacomo, L., Knudsen, B.R., and Andersen, A.H. (2004). The transducer domain is important for clamp operation in human DNA topoisomerase IIalpha. J. Biol. Chem. *279*, 1684–1691.

Olland, S., and Wang, J.C. (1999). 'Catalysis of ATP hydrolysis by two NH(2)-terminal fragments of yeast DNA topoisomerase II. J. Biol. Chem. *274*, 21688–21694.

Onoda, A., Hosoya, O., Sano, K., Kiyama, K., Kimura, H., Kawano, S., Furuta, R., Miyaji, M., Tsutsui, K., and Tsutsui, K.M. (2014). Nuclear dynamics of topoisomerase IIβ reflects its catalytic activity that is regulated by binding of RNA to the C-terminal domain. Nucleic. Acids. Res. *42*, 9005–9020.

Patel, S., Jazrawi, E., Creighton, A.M., Austin, C.A., and Fisher, L.M. (2000). 'Probing the interaction of the cytotoxic bisdioxopiperazine ICRF-193 with the closed enzyme clamp of human topoisomerase IIalpha. Mol. Pharmacol. *58*, 560–568.

Potterton, L., Agirre, J., Ballard, C., Cowtan, K., Dodson, E., Evans, P.R., Jenkins, H.T., Keegan, R., Krissinel, E., Stevenson, K., et al. (2018). CCP4i2: the new graphical user interface to the CCP4 program suite. Acta crystallogr. D Struct. Biol. *74*, 68–84.

Roca, J., Ishida, R., Berger, J.M., Andoh, T., and Wang, J.C. (1994). 'Antitumor bisdioxopiperazines inhibit yeast DNA topoisomerase II by trapping the enzyme in the form of a closed protein clamp. Proc. Natl. Acad. Sci. U S A. *91*, 1781–1785.

Roca, J., and Wang, J.C. (1992). 'The capture of a DNA double helix by an ATP-dependent protein clamp: a key step in DNA transport by type II DNA topoisomerases. Cell *71*, 833–840.

Roca, J., and Wang, J.C. (1994). 'DNA transport by a type II DNA topoisomerase: evidence in favor of a two-gate mechanism. Cell *77*, 609–616.

Rule, C.S., Patrick, M., and Sandkvist., M. (2016). Measuring in vitro ATPase activity for enzymatic characterization. JoVE *114*, 54305.

Schmidt, B.H., Burgin, A.B., Deweese, J.E., Osheroff, N., and Berger, J.M. (2010). 'A novel and unified two-metal mechanism for DNA cleavage by type II and IA topoisomerases. Nature *465*, 641–644.

Schmidt, B.H., Osheroff, N., and Berger, J.M. (2012). 'Structure of a topoisomerase II-DNA-nucleotide complex reveals a new control mechanism for ATPase activity. Nat. Struct. Mol. Biol. *19*, 1147–1154.

Sehested, M., Wessel, I., Jensen, L.H., Holm, B., Oliveri, R.S., Kenwrick, S., Creighton, A.M., Nitiss, J.L., and Jensen, P.B. (1998). 'Chinese hamster ovary cells resistant to the topoisomerase II catalytic inhibitor ICRF-159: a Tyr49Phe mutation confers high-level resistance to bisdioxopiperazines. Cancer Res. *58*, 1460–1468.

Shapiro, A.B., and Austin, C.A. (2014). A high-throughput fluorescence anisotropy-based assay for human topoisomerase II β-catalyzed ATP-dependent supercoiled DNA relaxation. Anal. Biochem. *448*, 23–29.

Skouboe, C., Bjergbaek, L., Oestergaard, V.H., Larsen, M.K., Knudsen, B.R., and Andersen, A.H. (2003). 'A human topoisomerase II alpha heterodimer with only one ATP binding site can go through successive catalytic cycles. J. Biol. Chem. *278*, 5768–5774.

Smith, C.V., and Maxwell, A. (1998). 'Identification of a residue involved in transition-state stabilization in the ATPase reaction of DNA gyrase. Biochemistry *37*, 9658–9667.

Stanger, F.V., Christoph, D., and Tilman, S. (2014). Structure of the N-terminal Gyrase B fragment in complex with ADP•Pi reveals rigid-body motion induced by ATP hydrolysis. PLoS. One. *9*, e107289.

Vagin, A., and Teplyakov, A. (1997). 'MOLREP: an automated program for molecular replacement. J. Appl. Crystallogr. *30*, 1022–1025.

van Dalen, E.C., Caron, H.N., Dickinson, H.O., and Kremer, L.C.M. (2011). Cardioprotective interventions for cancer patients receiving anthracyclines. Cochrane Database Syst. Rev. *6*. https://doi.org/10.1002/14651858.CD003917.pub4.

Vanden Broeck, A., Lotz, C., Drillien, R., Haas, L., Bedez, C., and Lamour, V. (2021). 'Structural basis for allosteric regulation of Human Topoisomerase IIalpha. Nat. Commun. *12*, 2962.

Walker, J.E., Saraste, M., Runswick, M.J., and Gay, N.J. (1982). Distantly related sequences in the alpha- and beta-subunits of ATP synthase, myosin, kinases and other ATP-requiring enzymes and a common nucleotide binding fold. Embo. J. *1*, 945–951.

Wang, J.C. (1998). 'Moving one DNA double helix through another by a type II DNA topoisomerase: the story of a simple molecular machine. Q. Rev. Biophys. *31*, 107–144.

Wei, H., Ruthenburg, A.J., Bechis, S.K., and Verdine, G.L. (2005). 'Nucleotide-dependent domain movement in the ATPase domain of a human type IIA DNA topoisomerase. J. Biol. Chem. *280*, 37041–37047.

Wells, N.J., and Fry, A.M. (1995). Fulvio guano, chris norbury, and ian D. Hickson. 1995. 'Cell cycle phase-specific phosphorylation of human topoisomerase IIα: evidence OF a role for protein KINASE C(*). J. Biol. Chem. *270*, 28357–28363.

Wendorff, T.J., Schmidt, B.H., Heslop, P., Austin, C.A., and Berger, J.M. (2012). 'The structure of DNA-bound human topoisomerase II alpha: conformational mechanisms for coordinating inter-subunit interactions with DNA cleavage. J. Mol. Biol. *424*, 109–124.

Wessel, I., Jensen, L.H., Jensen, P.B., Falck, J., Rose, A., Roerth, M., Nitiss, J.L., and Sehested, M. (1999). 'Human small cell lung cancer NYH cells selected for resistance to the bisdioxopiperazine topoisomerase II catalytic inhibitor ICRF-187 demonstrate a functional R162Q mutation in the Walker A consensus ATP binding domain of the alpha isoform. Cancer. Res. *59*, 3442–3450.

Wessel, I., Jensen, L.H., Renodon-Corniere, A., Sorensen, T.K., Nitiss, J.L., Jensen, P.B., and Sehested, M. (2002). 'Human small cell lung cancer NYH cells resistant to the bisdioxopiperazine ICRF-187 exhibit a functional dominant Tyr165Ser mutation in the Walker A ATP binding site of topoisomerase II alpha. FEBS. Lett. *520*, 161–166.

West, K.L., Turnbull, R.M., Willmore, E., Lakey, J.H., and Austin, C.A. (2002). 'Characterisation of the DNA-dependent ATPase activity of human DNA topoisomerase IIbeta: mutation of Ser165 in the ATPase domain reduces the ATPase activity and abolishes the in vivo complementation ability. Nucleic. Acids. Res. *30*, 5416–5424.

Wigley, D.B., Davies, G.J., Dodson, E.J., Maxwell, A., and Dodson, G. (1991). Crystal structure of an N-terminal fragment of the DNA gyrase B protein. Nature *351*, 624–629.

Williams, C.J., Headd, J.J., Moriarty, N.W., Prisant, M.G., Videau, L.L., Deis, L.N., Vishal, V., Keedy, D.A., Hintze, B.J., Chen, V.B., et al. (2018). 'MolProbity: more and better reference data for improved all-atom structure validation. Protein. Sci. *27*, 293–315.

Winter, G., Lobley, C.M.C., and Prince, S.M. (2013). Decision making in xia2. Acta Crystallogr. Sect. D Biol. Crystallogr. *69*, 1260–1273.

Winter, G., Waterman, D.G., Parkhurst, J.M., Brewster, A.S., Gildea, R.J., Gerstel, M., Fuentes-Montero, L., Vollmar, M., Michels-Clark, T., Young, I.D., Sauter, N.K., and Evans, G. (2018). DIALS: implementation and

evaluation of a new integration package. Acta Crystallogr. D Struct. Biol. *74*, 85–97.

Wu, C.C., Li, T.K., Farh, L., Lin, L.Y., Lin, T.S., Yu, Y.J., Yen, T.J., Chiang, C.W., and Chan, N.L. (2011). 'Structural basis of type II topoisomerase inhibition by the anticancer drug etoposide. Science *333*, 459–462.

Yang, X., Li, W., Prescott, E.D., Burden, S.J., and Wang, J.C. (2000). 'DNA topoisomerase IIbeta and neural development. Science *287*, 131–134.

Zheng, H., Cooper, D.R., Porebski, P.J., Shabalin, I.G., Handing, K.B., and Minor, W. (2017). CheckMyMetal: a macromolecular metal-binding validation tool. Acta Crystallogr. D Struct. Biol. *73*, 223–233.

STAR★METHODS

KEY RESOURCES TABLE

REAGENT or RESOURCE	SOURCE	IDENTIFIER
Bacterial and virus strains		
Rosetta 2 (DE3) pLysS	Sigma-Aldrich	Cat# 71403
Chemicals, peptides, and recombinant proteins		
2YT Autoinduction Media	Formedium	Cat# AIM2YT0210
His Trap HP Column (5mL)	Cytiva	Cat# 17524802
Hi Trap Heparin HP Column (5mL)	Cytiva	Cat# 17040701
HiLoad 16/600 Superdex 75	Sigma-Aldrich	Cat# GE28-9893-35
PACT Premier Screen	Molecular Dimensions	Cat# MD1-29
JCSG Plus Screen	Molecular Dimensions	Cat# MD1-37
Deposited data		
TOP2B ATPase Domain: AMPPNP	This Paper	PDB: 7QFO
TOP2B ATPase Domain: ADP	This Paper	PDB: 7QFN
TOP2B ATPase Domain: ADP and ICRF193	This Paper	PDB: 7ZBG
Oligonucleotides		
TOP2B (1–444) LIC Primer Forward: tacttccaatcca atgcaATGGCCAAGTCGGGTGGCTGC	This Paper	N/A
TOP2B (45–444) LIC Primer Forward: tacttccaatc caatgcaTCTGTTGAGAGAGTGTATCAGAAG	This Paper	N/A
TOP2B LIC Primer Reverse: ttatccacttccaatgttatt aTGAACACTTCTTATTCAGCTGAGTCTG	This Paper	N/A
TOP2B Alternative Splice Variant Forward: GGG CACTGACCTGGGTGACTCTTTTTGATCAGAAC AATGCTGC	This Paper	N/A
TOP2B Alternative Splice Variant Reverse: GCA GCATTGTTCTGATCAAAAAGAGTCACCCAGG TCAGTGCCC	This Paper	N/A
TOP2B E103A Forward: CCAGGTTTATACAAG ATCTTTGATGCAATTTTGGTTAATGCTGC	This Paper	N/A
TOP2B E103A Reverse: GCAGCATTAACCAAA ATTGCATCAAAGATCTTGTATAAACCTGG	This Paper	N/A
Software and algorithms		
MolProbity	Williams et al. (2018)	http://molprobity.biochem.duke.edu
Coot	Emsley et al. (2010)	https://www2.mrc-lmb.cam.ac.uk/personal/pemsley/coot/
REFMAC5	Murshudov et al. (2011)	https://www2.mrc-lmb.cam.ac.uk/groups/
MOLREP	Vagin and Teplyakov. (1997)	http://www.ccp4.ac.uk/html/molrep.html

RESOURCE AVAILABILITY

Lead contact
Further information and requests for resources and reagents should be directed to and will be fulfilled by the lead contact, Caroline Austin (caroline.austin@newcastle.ac.uk).

Materials availability
This study did not generate new unique reagents.

Data and code availability

Coordinates and structure factors have been deposited in the Protein Data Bank (http://www.ebi.ac.uk/pdbe/) with accession codes PDB: 7QFO (AMPPNP), PDB: 7QFN (ADP) and PDB: 7ZBG (ADP:ICRF193).

This paper does not report original code.

Any additional information required to reanalyze the data reported in this paper is available from the lead contact upon request.

EXPERIMENTAL MODEL AND SUBJECT DETAILS

Protein expression was performed using Rosetta 2 (DE3) pLysS cells (Novagen) transformed with the TOP2B plasmids. Expression and purification were done as described in method details.

METHOD DETAILS

Cloning

The coding sequence of human TOP2B ATPase domain (residues 45–444) was amplified from YEphTOP2βKLM(Meczes et al., 1997) by PCR and ligation independent cloning (LIC) using two primers, 5′-tacttccaatccaatgcaTCTGTTGAGAGAGTGTATCAGAAG -3′ and 5′-ttatccacttccaatgttattaTGAACACTTCTTATTCAGCTGAGTCTG-3′. The DNA sequence corresponding to the LIC sequence is represented in lowercase letters. The DNA sequence for the ATPase domain of human TOP2B was then inserted into the pET His6 MBP Asn10 TEV LIC cloning vector (1C) gifted from Scott Gradia (Addgene plasmid # 29,654; http://n2t.net/addgene:29654; RRID:Addgene_29,654) via LIC. The resulting plasmid (TOP2B 45–444 1C) encodes a fusion protein with an N-terminal 6-His tag followed by maltose binding protein (MBP) and human TOP2B ATPase with a tobacco etch virus (TEV) protease cleavage site in between. The full-length ATPase domain starting at amino acid 1 (TOP2B 1–444) of TOP2B was also cloned as detailed above by LIC, from a plasmid containing residues 1–476 pGEX1λT (unpublished). The reverse LIC primer was the same as used for TOP2B 45–444 and the forward LIC primer was 5′-TACTTCCAATCCAATGCAATGGCCAAGTCGGGTGGCTGC-3′. Site directed mutagenesis (QuikChange II Site-Directed Mutagenesis Kit, Agilent) of plasmid TOP2B 1–444 was used to insert the additional five amino acids in the alternative splice variant of human TOP2B using the following primers: 5′-GGGCACTGACCTGGGTGACTCTTTTTGATCAGAA CAATGCTGC-3′ and 5′-GCAGCATTGTTCTGATCAAAAAGAGTCACCCAGGTCAGTGCCC-3′. In addition, the two catalytic inactive control proteins E103A in TOP2B 45–444 and E103A in TOP2B 1–444 were generated via site directed mutagenesis with the primers 5′-CCAGGTTTATACAAGATCTTTGATGCAATTTTGGTTAATGCTGC-3′ and 5′-GCAGCATTAACCAAAATTGCATCAAAGATCTTGT ATAAACCTGG-3′.

Expression and purification

Transformed Rosetta 2 (DE3) pLysS cells were grown in 2YT autoinduction media (Formedium) at 37°C for 4 h and then the temperature was reduced to 20°C for overnight incubation. The following morning, cells were harvested by centrifugation and lysed by sonication in 50 mM Tris HCl (pH 8.0), 150 mM NaCl. The fusion protein (6His-MBP-human TOP2B ATPase) was first purified over a Ni^{2+} affinity column (Cytiva Ni^{2+} Sepharose High Performance affinity resin) and then the 6His-MBP tag was removed by TEV digestion. Human TOP2B ATPase was further purified with a heparin column (Cytiva Heparin Sepharose High Performance resin) and 6His-MBP was removed by passing the heparin eluate through a second round of Ni^{2+} affinity. The flow through was collected and concentrated with an MWCO 30 kDa device (Millipore). The final purification step was gel filtration with 20 mM Tris HCl (pH 8.0), 150 mM NaCl on a Hiload 16/60 Superdex 75 size exclusion column. Peak fractions containing human TOP2B ATPase were concentrated to 10 mg/mL for use in crystallography.

Crystallization

To obtain crystals of human TOP2B ATPase (45–444) bound to AMPPNP, 1 mM AMPPNP (Sigma) and 5 mM $MgCl_2$ was added to the protein stock. Crystallization screens by sitting drop vapor diffusion were set up. 300 nL protein stock was mixed with 600 nL crystallization buffer [0.1 M PCTP (pH 7.0), 25% w/v PEG 1500] (PACT, C4, Molecular Dimensions) using the Mosquito crystallization robot (TTP Labtech) in two-well MRC plates at 20°C. Crystals (≈200 × 50 × 50 μm) appeared and reached maximal size within 48 h, after which they were harvested in crystallization solution supplemented with 20% PEG400 (v/v) for cyro-protection and flash cooled in liquid nitrogen.

Human TOP2B ATPase (45–444) crystals bound to ADP, and ADP:ICRF193 crystals were also obtained by sitting drop vapor diffusion. 5 mM ADP (Sigma), 5 mM $MgCl_2$ and 0.1 mg ICRF193 (Sigma) for ADP:ICRF193 crystals were added to the protein stock. Crystals were grown at 20°C using in MRC plates two well by mixing 300 nL of protein solution with 600 nL crystallization buffer [0.2 M ammonium sulfate, 0.1 M bis tris (pH 5.5), 25% w/v PEG 3350] (JCSG+, H7, Molecular Dimensions) using the Mosquito crystallization robot (TTP Labtech). Crystals grew to the maximal size of ≈50 × 20 × 10 μm within 1 week, after which they were harvested in crystallization solution supplemented with 20% PEG400 (v/v) for cyro-protection and flash-cooled in liquid nitrogen.

Data collection and processing

Diffraction data for all crystals were collected at diamond beamline I03 with unattended data collection using the native data collection strategy https://www.diamond.ac.uk/Instruments/Mx/I03/I03-Manual/Unattended-Data-Collections/Experiment-Types.html. Data were processed at diamond with the automated pipeline xia2(Winter et al., 2013) with dials(Winter et al., 2018). The data were scaled with Aimless(Evans and Murshudov 2013) and the space groups were confirmed with Pointless(Evans and Murshudov 2013). 5% of the data were randomly selected for R_{free} calculations. The processing statistics are presented in Table 1.

Phase problem and model building

The phase problem was solved for the human TOP2B ATPase AMPPNP structure on CCP4 cloud Newcastle (Potterton et al., 2018) by molecular replacement using molrep or phaser (Vagin and Teplyakov 1997; McCoy et al., 2007). The human TOP2A ATPase (83% sequence identity to human TOP2B) was used as a search model for AMPPNP-bound (PDB code 1ZXM). In turn the AMPPNP model (PDB code 7QFO) was used to solve the ADP-bound structures. The models were improved by iterative cycles of manual model building in COOT (Emsley et al., 2010) and refined using refmac (Murshudov et al., 2011). The models were validated using COOT (Emsley et al., 2010) validation tools and molprobity (Williams et al., 2018). Metal type, co-ordination and bond distances were validated using check my metal (Zheng et al., 2017). Structural figures were generated using Pymol (The Pymol Molecular Graphics System, Version 2.0 Schrödinger, LLC). Structures were superposed with the Secondary Structure Matching or Least Squares Fitting tool in COOT. Other software used were from the CCP4 suite (Potterton et al., 2018).

ATPase activity assays

ATPase activity assays were performed using BioMOL green (Enzo)(Rule et al. 2016). The assays were performed in a reaction mixture containing 50 mM Tris HCl pH 7.5, 50 mM KCl, 10 mM $MgCl_2$, and 0.1 mM ATP (unless otherwise stated). TOP2B ATPase domain proteins were then added to start the reaction and incubated at 37°C for 30 min. The concentrations of TOP2B were varied as detailed in figure legends. For DNA stimulation experiments, 4 μg pBR322 DNA was added prior to TOP2B. 50 μL of the reaction was then transferred to a microtitre plate containing 100 μL BioMOL green to terminate the experiment. After 20 min the absorbance was measured at 655 nm. Reactions containing no enzyme were performed to generate a background reading of inorganic phosphate and were subtracted from the experimental results. The inorganic phosphate released was then calculated based on the absorbance standard curve established by phosphate standards. The kinetic parameters K_m and V_{max} were calculated from the Lineweaver-Burk plots using GraFit. All experiments were repeated at least three times.

QUANTIFICATION AND STATISTICAL ANALYSIS

Data reported for the ATP hydrolysis activity assays are the mean of three experimental replicates. Error bars represent one SD from the mean. Data were processed in GraphPad Prism 4. Statistical analysis was performed by a one-way ANOVA and Tukey's Multiple Comparison Test or a two-way ANOVA and Bonferroni post-tests (as detailed in figure legend).

Structure, Volume *30*

Supplemental Information

A comprehensive structural analysis of the ATPase domain of human DNA topoisomerase II beta bound to AMPPNP, ADP, and the bisdioxopiperazine, ICRF193

Elise M. Ling, Arnaud Baslé, Ian G. Cowell, Bert van den Berg, Tim R. Blower, and Caroline A. Austin

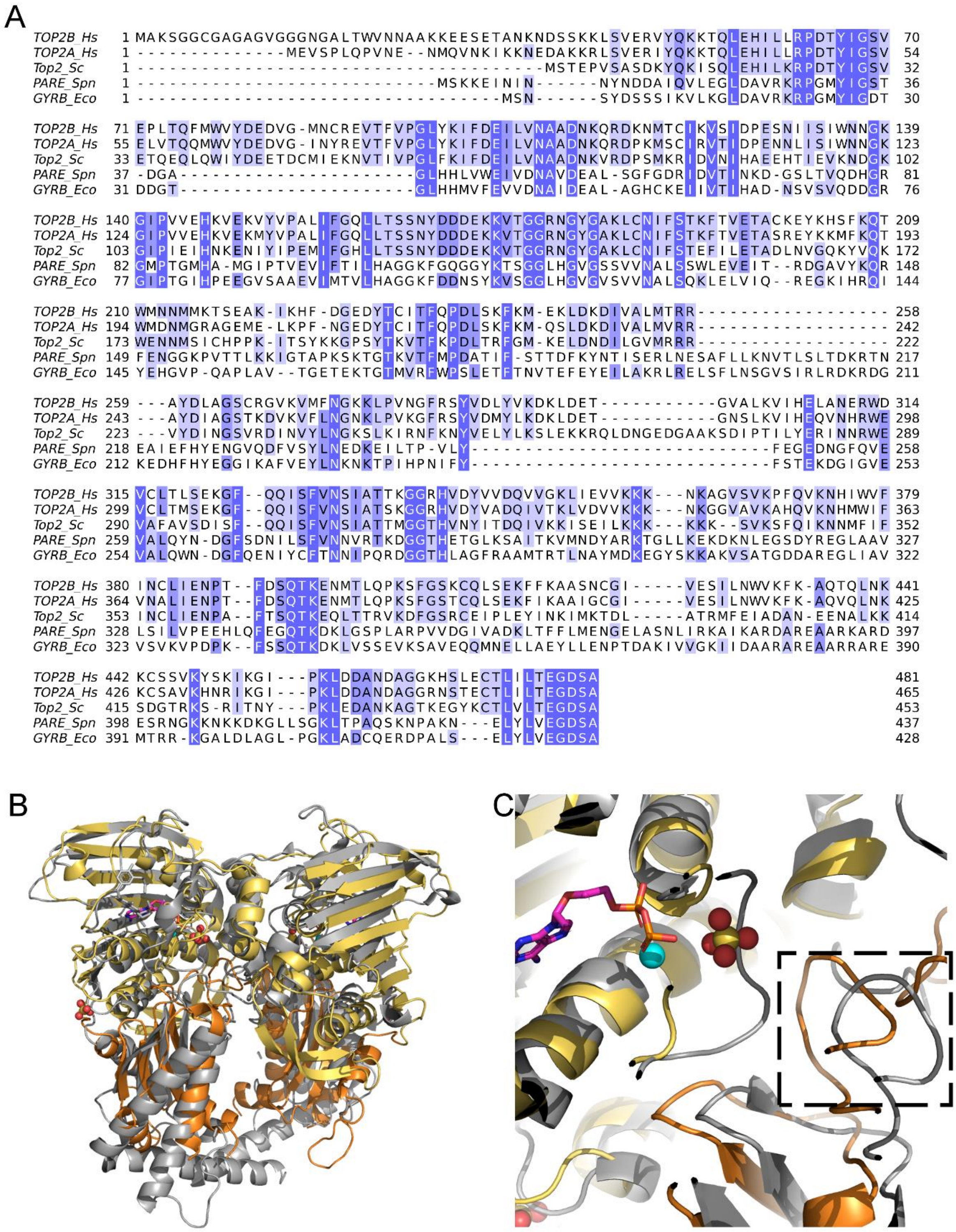

Supplementary Figure 1: Clustal Alignment and Crystal Structures of GyrB, Related to Figure 1

(**A**) Clustal alignment comparing Gyrase B, ParE with eukaryotic type II topoisomerases (human TOP2B, human TOP2A, yeast TOP2). (**B-C**) Superposition of Gyrase B (grey) (pdb 1KIJ) onto human TOP2B:ADP ATPase domain (yellow and orange). The transducer helices towards the end of the ATPase domain in GyrB are in a more open conformation compared to human TOP2B. Moreover, the QTK loop in GyrB (Panel C boxed region) is further from the active site in comparison to human TOP2B.

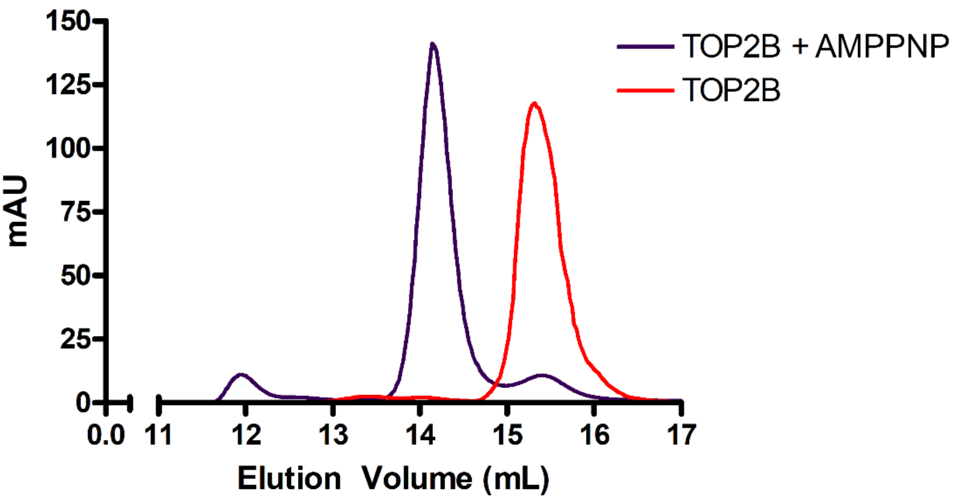

Supplementary Figure 2: Analytical Gel Filtration of the ATPase Domain of Human TOP2B, Related to Figure 1

Analytical gel filtration of TOP2B (45-444) in the absence (red) and presence (purple) of AMPPNP. TOP2B protein injected onto a Superdex 200 Increase 10/300 GL column with 20 mM Tris HCl (pH 8.0), 150 mM NaCl. Blue line indicates UV absorbance. In the absence of AMPPNP, TOP2B elutes at ~15.3 mL corresponding to an apparent Mw of 47 kDa, equivalent to the theoretical size (45 kDa), whereas upon addition of AMPPNP, the protein elutes earlier at ~14.1 mL, corresponding to an apparent Mw of 78 kDa.

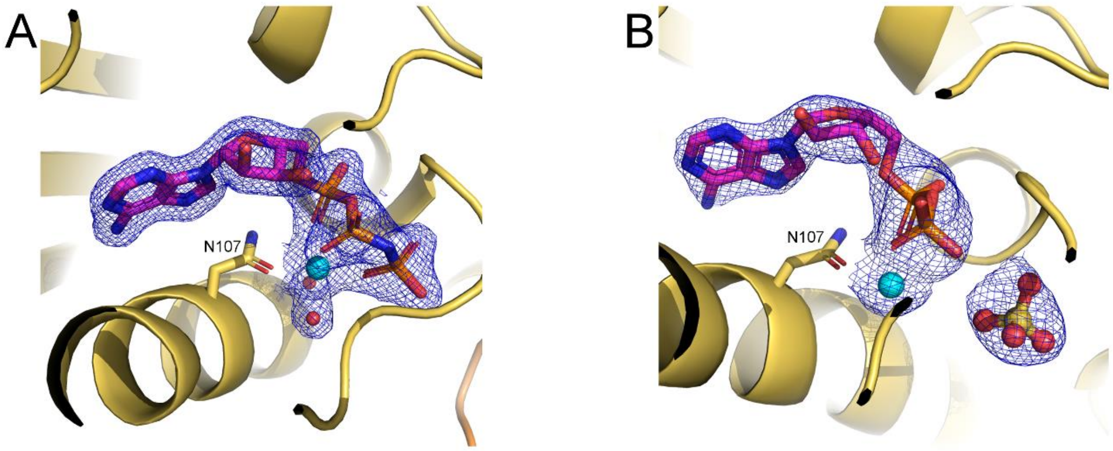

Supplementary Figure 3: Omit Maps for Bound Nucleotides, Related to Figure 2 (A) and Figure 3 (B)

The 2 Fo – Fc maps electron density maps for the 1.9 Å resolution AMPPNP complex (**A**), and the 2.6 Å resolution ADP complex (**B**) represented by blue mesh. The maps are contoured at levels of 1.5 σ and carved at 1.6 Å. Mg^{2+} shown in cyan and two water molecules shown in A as red spheres and sulphate ion represented by red and yellow spheres in B. Residue N107 shown as sticks.

Supplementary Table 1: Summary of selected homologous PDB entries relevant to this study and related to Figure 1B, Figure 5, Figure 6, STAR Methods and Supplemental Figure 1.

Protein Source	PDB code	Resolution (Å)	Conformation	Nucleotide	PMID	Reference
GyrB43 *E.coli*	N/A	2.5	closed	AMPPNP	1646964	Wigley et al, 1991
GyrB43 *E.coli*	4WUB	1.75	restrained	AMPPNP	25849408	Hearnshaw et al. 2015
GyrB43 *E.coli*	4PU9	2.4	semi-open Pre-hydrolysis	ADP.BeF3	25202966	Stanger et al. 2014
GyrB43 *E.coli*	4PRX	1.8	open Post-hydrolysis	ADP.Pi	25202966	Stanger et al. 2014
GyrB43 *E.coli*	4PRV	2.0	Closed product	ADP	25202966	Stanger et al. 2014
GyrB43 *E.coli*	1EI1	2.3	closed	AMPPNP	10734094	Brino et al. 2000
GyrB43 *E.coli*	1KIJ	2.3	open	(novobiocin and formic acid)	11850422	Lamour et al. 2002
ParE *E.Coli*	1S16	2.1	closed	AMPNP	15105144	Bellon et al. 2004
ParE *S. pneumoniae*	5J5P	2.83	closed	AMPNP	29968711	Laponogov, et al, 2018
ParE S. pneumoniae	5J5Q	2.83	closed	AMPNP	29968711	Laponogov, et al, 2018
Archaeal GHL enzyme topoisomerase VI topoVI-B *S. shibatae*	1MU5	2.0	relaxed	Apo	12505993	Corbett & Berger 2003
topoVI-B *S. shibatae*	1MX0	2.3	restrained	AMPNP	12505993	Corbett & Berger 2003
topoVI-B *S. shibatae*	1Z5B	2.0	restrained	$ADP.AlF_4^-$	15939019	Corbett & Berger 2005
topoVI-B *S. shibatae*	1Z5C	2.2	restrained	ADP.Pi	15939019	Corbett & Berger 2005
topoVI-B S. shibatae	1Z5A	2.2	Restrained (dimer)	ADP	15939019	Corbett & Berger 2005
topoVI-B S. shibatae	1Z59	2.1	Relaxed (monomer)	ADP	15939019	Corbett & Berger 2005
TOP2 *S.Cerevisiae*	1PVG	1.8	restrained	AMPPNP	12963818	Classen et al. 2003
TOP2A *H. sapiens*	1ZXM	1.87	restrained	AMPPNP	16100112	Wei et al. 2005
TOP2A *H. sapiens*	1ZXN	2.51	open	ADP	16100112	Wei et al. 2005
TOP2A *H. sapiens*	4R1F	-	open	ADP.SO4	25202966	Stanger et al. 2014
TOP2 *S.Cerevisiae*	4GFH	4.41		AMPPNP	23022727	Schmidt, Osheroff and Berger 2012
